# Emergence, Retention and Selection: A Trilogy of Origination for Functional *De Novo* Proteins from Ancestral LncRNAs in Primates

**DOI:** 10.1371/journal.pgen.1005391

**Published:** 2015-07-15

**Authors:** Jia-Yu Chen, Qing Sunny Shen, Wei-Zhen Zhou, Jiguang Peng, Bin Z. He, Yumei Li, Chu-Jun Liu, Xuke Luan, Wanqiu Ding, Shuxian Li, Chunyan Chen, Bertrand Chin-Ming Tan, Yong E. Zhang, Aibin He, Chuan-Yun Li

**Affiliations:** 1 Beijing Key Laboratory of Cardiometabolic Molecular Medicine, Institute of Molecular Medicine, Peking University, Beijing, China; 2 Center for Bioinformatics, National Laboratory of Protein Engineering and Plant Genetic Engineering, College of Life Sciences, Peking University, Beijing, China; 3 FAS Center for Systems Biology & Howard Hughes Medical Institute, Harvard University, Cambridge, Massachusetts, United States of America; 4 Peking-Tsinghua Center for Life Sciences, Beijing, China; 5 Key Laboratory of Zoological Systematics and Evolution, Institute of Zoology, Chinese Academy of Sciences, Beijing, China; 6 Molecular Medicine Research Center, Chang Gung University, Tao-Yuan, Taiwan; University of Michigan, UNITED STATES

## Abstract

While some human-specific protein-coding genes have been proposed to originate from ancestral lncRNAs, the transition process remains poorly understood. Here we identified 64 hominoid-specific *de novo* genes and report a mechanism for the origination of functional *de novo* proteins from ancestral lncRNAs with precise splicing structures and specific tissue expression profiles. Whole-genome sequencing of dozens of rhesus macaque animals revealed that these lncRNAs are generally not more selectively constrained than other lncRNA loci. The existence of these newly-originated *de novo* proteins is also not beyond anticipation under neutral expectation, as they generally have longer theoretical lifespan than their current age, due to their GC-rich sequence property enabling stable ORFs with lower chance of non-sense mutations. Interestingly, although the emergence and retention of these *de novo* genes are likely driven by neutral forces, population genetics study in 67 human individuals and 82 macaque animals revealed signatures of purifying selection on these genes specifically in human population, indicating a proportion of these newly-originated proteins are already functional in human. We thus propose a mechanism for creation of functional *de novo* proteins from ancestral lncRNAs during the primate evolution, which may contribute to human-specific genetic novelties by taking advantage of existed genomic contexts.

## Author Summary

Although gene duplication has been believed as a predominant mechanism for creating new genes, recent reports suggested that new proteins could evolve “*de novo*” from non-coding DNA regions. These *de novo* genes are also named as “motherless” genes due to their lack of ancestral proteins as precursors, while recently we and others found that lncRNAs may represent an intermediate stage of their origination. To further elucidate this lncRNA-protein transition process, here we identified 64 hominoid-specific *de novo* genes and report a new mechanism for the origination of functional *de novo* proteins from ancestral non-coding transcripts: These non-coding “precursors” are generally not more selectively constrained than other lncRNA loci; and the existence of these *de novo* proteins is not beyond anticipation under neutral expectation; however, population genetics study in 67 human individuals and 82 macaque animals revealed signatures of purifying selection on these genes specifically in human population, indicating a proportion of these newly-originated proteins are already functional in human. We thus propose a mechanism for creation of functional *de novo* proteins from ancestral lncRNAs during the primate evolution.

## Introduction

Although it is a generally accepted notion that gene duplication is the major way to create new genes [1–3], numerous cases have been reported in recent years demonstrating in multiple different species the creation of new proteins out of ancestral non-coding DNAs [4–18]. Recent studies further suggest that this *de novo* mechanism for gene origination may account for a significant proportion of new genes [2,14] and contribute to lineage-specific genetic novelties [18–20].

Currently, several comparative transcriptome studies have proposed that a proportion of *de novo* genes may originate from ancestral long non-coding RNAs (lncRNAs) [7,16,21], while the evolutionary mechanism underlying this lncRNA-protein transition remains elusive. First, it is unknown whether the differences in functional significance of lncRNAs, or some other sequence features, could explain the biased origination process of *de novo* genes from a specific subset of lncRNAs. Specifically, given that ancestral lncRNAs have precise splicing structures and tissue expression profiles similar to those of *de novo* proteins in human [16], it is unclear whether they have already obtained certain biological functions on the RNA level: one reason for us to hypothesize that functional non-coding genes may be favorable precursors is because they might survive longer during evolution, providing a wider time window for the emergence and stabilization of ORF, assuming that the emergence of the protein coding part does not interfere with the original function.

Second, it is unclear whether the human *de novo* genes have gained functional significance. Although it has been an established notion that *de novo* protein-coding genes could have important functions in *Drosophila* [17,18,22], the functional significance of *de novo* genes in hominoid lineage is still controversial–given the smaller effective population size in hominoids [23], the detection of these genes may be largely due to the weaker selection for removing the translational noises. Actually, for the dozens of human-specific *de novo* genes identified, only a few genes were linked to human diseases and regulations by circumstantial evidence [11,24–26]. While functional studies with transgenic monkeys could potentially characterize the functions of these hominoid-specific proteins, it is still technically challenging and could not provide a global view of the extent to which these genes are functional. Alternatively, a comparative population genetics approach, *i*.*e*. characterizing polymorphisms in the gene locus and comparing the pattern to that of the orthologous region in a closely related species, could provide evolutionary clues to the functional significance of the *de novo* genes.

We thus performed a population genetics study in human and rhesus macaque to interrogate the origin and functional significance of these newly-originated *de novo* protein-coding genes. We noted that these proteins in human seem to have originated from ancestral GC-rich lncRNAs. Although these lncRNAs generally are not more selectively constrained than other lncRNA loci, and the existence of these newly-originated proteins is not beyond anticipation under neutral expectation, our results showed that at least a proportion of these *de novo* proteins should have acquired protein-level functions, based on the signatures of purifying selection detected specifically in human populations. We thus propose a mechanism for creation of functional *de novo* proteins from ancestral lncRNAs during the primate evolution.

## Results

### Identification of 64 hominoid-specific *de novo* proteins originated from lncRNAs

**Fig 1:**
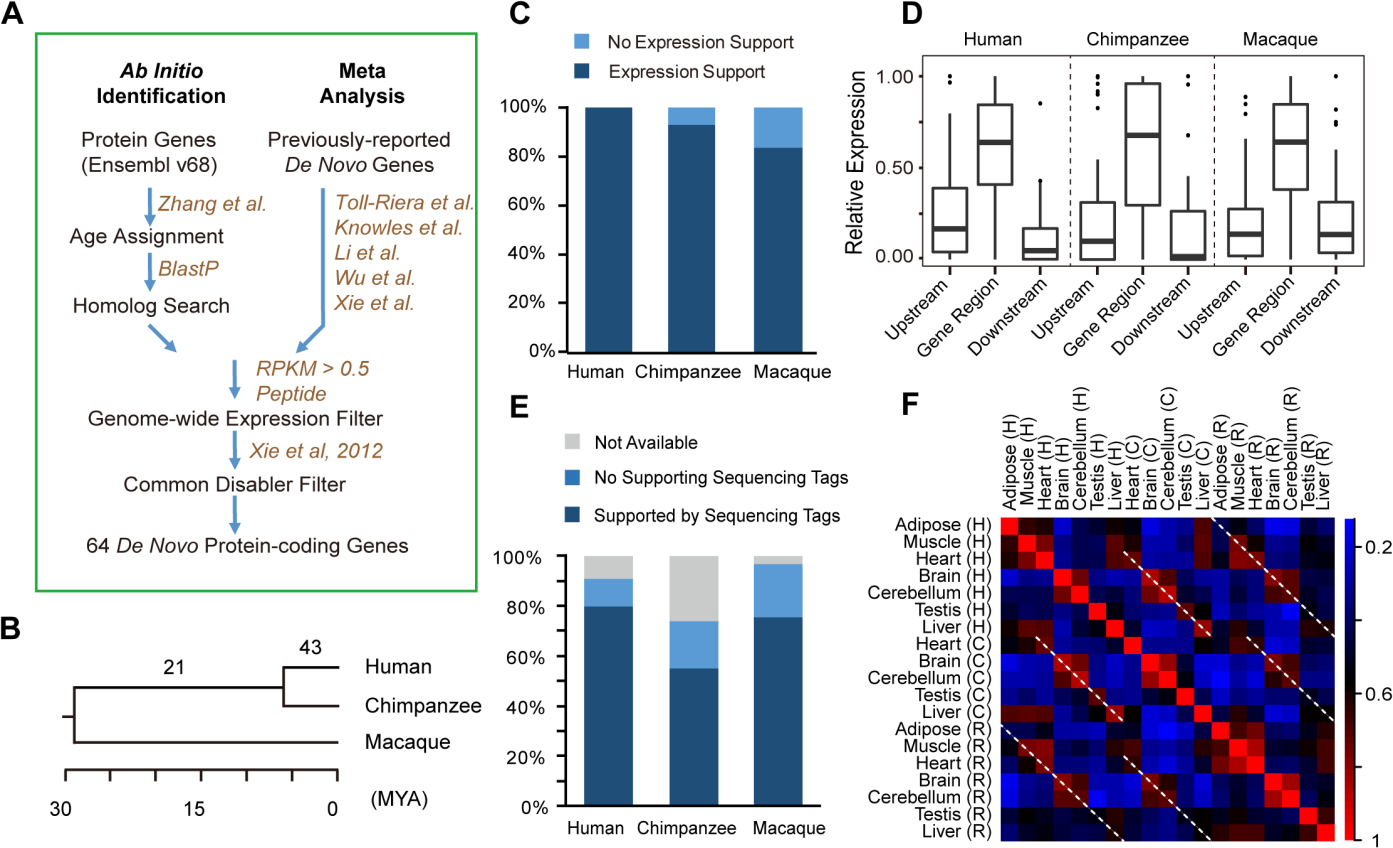
*De novo* protein-coding genes originating from lncRNAs. (**A**) Computational pipeline for *ab inito* identification and meta-analysis of *de novo* genes in the hominoid lineage. (**B**) Number of *de novo* genes on the phylogenetic tree, with the branch length proportional to the divergence time. (**C**) Stacked histogram showing the percentage of *de novo* gene orthologs that also show expression in chimpanzee or rhesus macaque. (**D**) Boxplot showing relative expression levels of the transcripts and their nearby regions corresponding to *de novo* genes (orthologs) in human (chimpanzee or macaque). The nearby regions are defined as upstream and downstream regions with equal length to the corresponding genes. For each region, the relative expression was calculated by normalizing the expression level of this region with the sum of the expression levels of the genic region and the nearby regions. (**E**) Percentage of splicing junctions with supporting RNA-Seq reads in human, chimpanzee and rhesus macaque. (**F**) For each pair of tissues, *Spearman* correlation coefficients were computed separately, and the extent of tissue-specific differences in *de novo* gene expressions are shown (based on the color scale). Dotted lines highlight parallel comparisons between two different species.

To interrogate the genesis and functional implications of *de novo* proteins in primates, we firstly performed a comprehensive survey for newly-originated *de novo* protein-coding genes in the hominoid lineage. We devised a genome-wide pipeline integrating *ab initio* identifications [16] and meta-analysis of public datasets [9–11,13,16] (**Fig 1A; Materials and Methods**). Briefly, we first inferred the locus ages on the basis of the syntenic genomic alignment generated by UCSC, and only retained human genes with high-quality alignments in the out-group species (**Discussion**). With this approach, the potential bias in *de novo* gene identification introduced by blast-like alignments is well controlled [27]. Then, for each locus, the existence of the ORF in multiple out-group species was inferred separately (**Materials and Methods)**. Candidate *de novo* genes were then identified based on age assignments of ORFs, by summing up the information on the presence and absence of orthologous ORFs in vertebrate phylogeny with the principle of parsimony [2,16]. We further performed sequence similarity study to analyze these candidates against all annotated human proteins, further verifying that they originated through *de novo* evolution, rather than other mechanisms such as gene duplication (**Materials and Methods)**.

The resulting 56 candidates, together with 99 literature-documented primate-specific *de novo* genes [9–11,13,16], were then subjected to additional inclusion criteria (**Materials and Methods)**. Consequently, only genes with 1) reliable evidence for transcriptional and translational activities in human (**S1 Fig; Materials and Methods**), and 2) detectable common ancestral “disablers”, disrupting the ORFs in all out-group species at the same sequence position [9,11], as indication for newly-created but not old dying genes, were included (**Fig 1A; Materials and Methods**).

In total, 64 protein-coding genes were identified with recent origination in the hominoid lineage through *de novo* evolution (**Fig 1B; Tables 1** and **S1**), with 43 encoding human-specific proteins (**Class I**, the younger proteins), and another 21 encoding similar proteins in human and chimpanzee but not in rhesus macaque (**Class II**, the older proteins).

**Table 1:** Basic information of 64 *de novo* genes in hominoid lineage.

Gene ID^#^	Age^%^	Length^&^	Expression^$^	Peptides	Source
ENSG00000178803	H	159	Kidney, 4	7 [28]	[9,16] [*]
ENSG00000204626	H	163	Cerebellum, 7	8 [28]	[9,13,16] [*]
ENSG00000145063	H	174	Brain, 1	12 [28]	[16] [*]
ENSG00000172927	H	313	Breast, 3	14 [28–30]	[16] [*]
ENSG00000177822	H-C	148	Adipose, 4	7 [28]	[16] [*]
ENSG00000179522	H-C	230	Prostate, 5	9 [28]	[16] [*]
ENSG00000215071	H-C	121	Testes, 14	2 [16]	[16] [*]
ENSG00000182457	H-C	135	Ovary, 17	3 [28]	[16] [*]
ENSG00000174407	H-C-O	99	Heart, 5	6 [28]	[16] [*]
ENSG00000203930	H	103	Cerebellum, 2	2 [16]	[16] [*]
ENSG00000204091	H-C-O	100	Testes, 3	3 [28]	[16] [*]
ENSG00000204666	H-C	122	Brain, 2	8 [28]	[16] [*]
ENSG00000204674	H-C	123	Cerebellum, 13	10 [28]	[16] [*]
ENSG00000212736	H-C-O	115	Adrenal, 16	8 [28]	[16] [*]
ENSG00000167747	H-C-O	117	Testes, 17	11 [28–30]	[16] [*]
ENSG00000214112	H-C-O	72	Heart, 2	3 [28]	[16] [*]
ENSG00000214130	H-C	149	Heart, 2	5 [16]	[16] [*]
ENSG00000118267	H	423	Colon, 17	33 [28]	[16] [*]
ENSG00000215458	H	302	Blood, 5	18 [28]	[16] [*]
ENSG00000215494	H	152	Breast, 1	12 [28]	[16] [*]
ENSG00000215848	H	161	Brain, 3	6 [28]	[16] [*]
ENSG00000221953	H	237	Brain, 1	15 [28]	[16] [*]
ENSG00000221891	H-C-O	157	Testes, 4	13 [28]	[16] [*]
ENSG00000221899	H	166	Lymph_node, 10	17 [28]	[16] [*]
ENSG00000205056	H	121	Blood, 1	2 [28]	[9]
ENSG00000198547	H	194	Brain, 2	13 [28]	[11]
ENSG00000136242	H	128	Testes, 16	4 [28]	[13]
ENSG00000162968	H	151	Brain, 3	4 [28]	[13]
ENSG00000175913	H	147	Cerebellum, 2	9 [28]	[13]
ENSG00000176833	H	126	Testes, 1	7 [28]	[13]
ENSG00000176911	H	134	Breast, 1	5 [28]	[13]
ENSG00000180838	H	131	Prostate, 3	4 [28]	[13]
ENSG00000187488	H	221	Testes, 17	2 [13]	[13]
ENSG00000196273	H	105	Testes, 1	10 [28]	[13]
ENSG00000197916	H	129	Adipose, 16	3 [28]	[13]
ENSG00000204079	H	141	Adrenal, 1	6 [28]	[13]
ENSG00000204292	H	150	Testes, 1	2 [13]	[13]
ENSG00000204380	H	155	Cerebellum, 10	6 [28]	[13]
ENSG00000205373	H	219	Testes, 13	4 [13]	[13]
ENSG00000205557	H	149	Cerebellum, 3	2 [28]	[13]
ENSG00000205965	H	175	Kidney, 2	4 [28]	[13]
ENSG00000206028	H	164	Brain, 5	8 [28]	[13]
ENSG00000206096	H	127	Testes, 4	5 [28]	[13]
ENSG00000206110	H	129	Brain, 1	8 [28]	[13]
ENSG00000206113	H	213	Testes, 1	13 [28]	[13]
ENSG00000212693	H	131	Thyroid, 6	3 [13]	[13]
ENSG00000214780	H	195	Brain, 1	3 [28]	[13]
ENSG00000218478	H	158	Kidney, 16	2 [13]	[13]
ENSG00000223857	H	131	Brain, 12	7 [28]	[13]
ENSG00000224013	H	164	Cerebellum, 6	8 [28]	[13]
ENSG00000225021	H	144	Liver, 7	4 [28]	[13]
ENSG00000225860	H	175	Brain, 1	8 [28]	[13]
ENSG00000225917	H	269	Brain, 15	15 [28]	[13]
ENSG00000230294	H	119	Testes, 1	8 [28]	[13]
ENSG00000235766	H	142	Lung, 12	4 [28]	[13]
ENSG00000236314	H	156	Testes, 17	10 [28]	[13]
ENSG00000260456	H-C-G	158	Testes, 15	6 [28]	[*]
ENSG00000149443	H-C	151	Testes, 1	11 [28]	[*]
ENSG00000167159	H-C-O	157	Cerebellum, 7	1 [28]	[*]
ENSG00000008517	H-C	188	Kidney, 17	3 [28]	[*]
ENSG00000183250	H-C-O	204	Brain, 7	7 [28]	[*]
ENSG00000244291	H-C	216	Testes, 17	9 [28]	[*]
ENSG00000205913	H-C	107	Brain, 3	4 [28]	[*]
ENSG00000221990	H-C-G-O	119	Testes, 13	15 [28]	[*]

^**#**^Gene IDs from the original studies

^**%**^H: human, C: chimpanzee, G: gorilla and O: Orangutan

^**&**^Stop codons are excluded

^**$**^Tissue in which the *de novo* gene is most highly-expressed and the number of tissues (up to 17 tissues) in which the *de novo* gene is expressed (RPKM>0.5)

*This study.

The transcript structure and expression of these genes at the transcriptional or translational levels in human are strongly supported by public genomics data. The transcriptional structure for all of these genes were supported by full-length mRNA or spliced EST evidence (**S2 Table**), with 88% of the splicing junctions also supported by short RNA-Seq reads (**Fig 1E; Materials and Methods**); the full-length transcript structure for 17 of these genes were also verified by the Iso-Seq data, generated recently through the PacBio transcriptome sequencing (**S2 Table; Materials and Methods**). In addition, the protein expressions for all of these genes were supported by large-scale mass spectrometry studies in human (**Tables 1** and **S2; Materials and Methods**).

To infer the transcriptional capacity of the 64 *de novo* genes in the common ancestor of human and closely related species, we performed cross-species transcriptome analysis in human, chimpanzee and rhesus macaque. First, we found that 83.9% of the 64 genes, and 92.9% of the 43 human-specific genes transcribed in at least one tissue in rhesus macaque or chimpanzee as lncRNAs (**Fig 1C**), with the expression levels significantly higher than the background expression levels (**S1 Fig**; **Materials and Methods**). Second, the expression levels of the genic regions relative to upstream and downstream regions were comparable among the three species (**Fig 1D**), and the majority of human splicing junctions were also detectable in chimpanzee and rhesus macaque orthologous regions (**Fig 1E**). Third, the non-coding orthologs of human *de novo* genes in rhesus macaque and chimpanzee also show tissue expression profiles similar to human (**Fig 1F**). The inter-species similarity of tissue expression profiles was further supported by clustering analysis, with the same tissue types from different species clustered together (**S2 Fig** and **S3 Table**).

By the parsimony principle, we conclude that the transcription structure and expression profile of these *de novo* genes had been shaped in the common ancestor prior to the acquisition of coding potential in the human lineage. It is thus interesting to investigate whether these lncRNA precursors with precise splicing structures and tissue expression profiles have already obtained certain biological functions on the RNA level, and may thus represent favorable precursors for new *de novo* proteins.

### lncRNA precursors are not more selectively constrained than other lncRNA loci

Because lncRNAs could have a variety of functions, not all of which can be easily assayed, as an alternative, we sought an evolutionary approach by quantifying the level of selective constraints in the orthologous lncRNA loci of these *de novo* genes in rhesus macaque as a proxy for determining the functional status in the ancestor. This assumes that the selective constraints on these loci have remained unchanged in the macaque lineage since it had a common ancestor with human.

**Fig 2:**
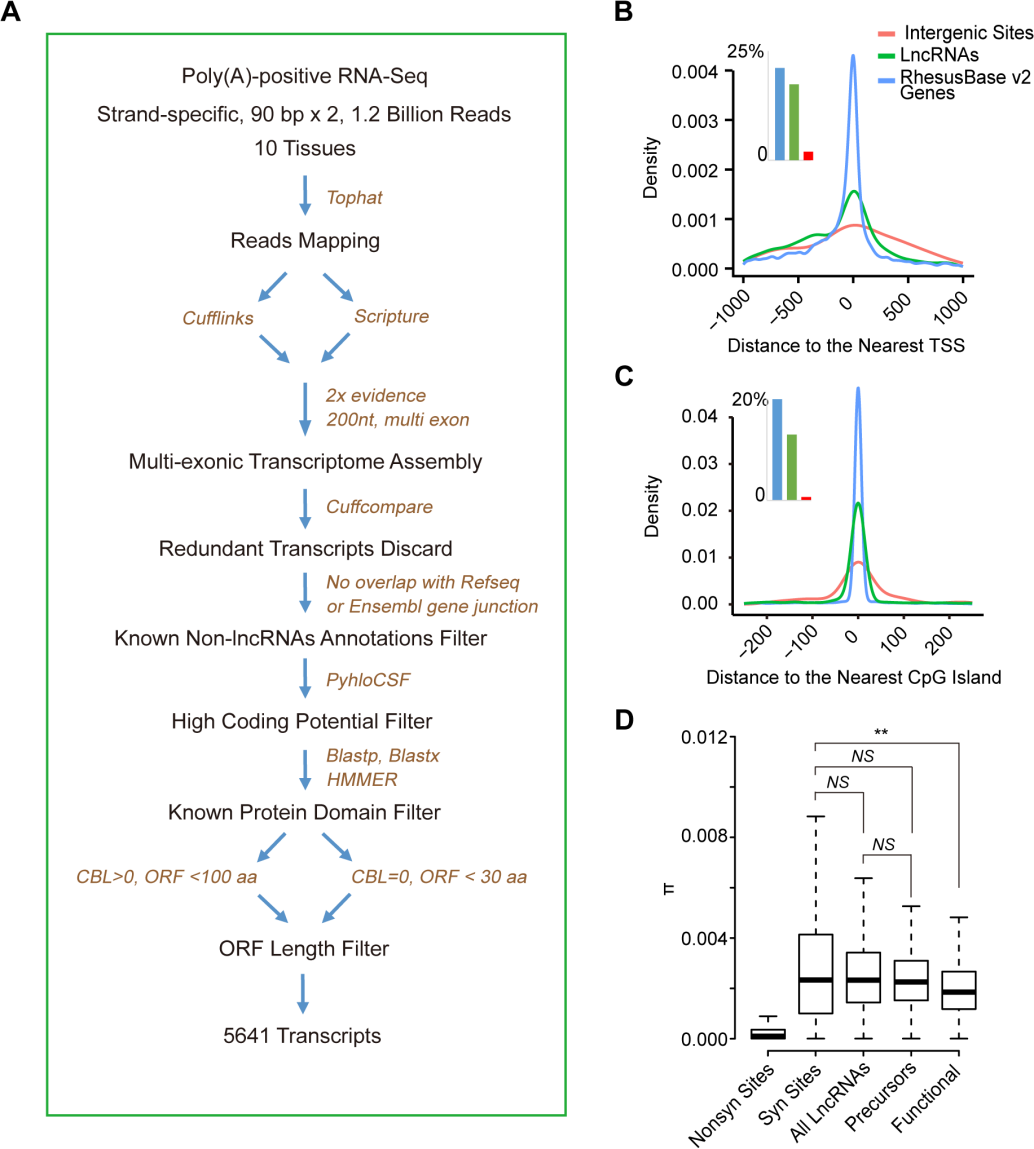
*De novo* proteins originate from lncRNAs precursors irrespectively of their functional status at RNA level. **(A)** Flow chart showing the computational pipeline of lncRNAome identification in rhesus macaque. (**B, C**) For lncRNAs identified in this study, the distribution of distances between 5’ end of lncRNAs and the nearest annotated transcript start site (TSS) (**B**) or CpG island (**C**) are shown. The numbers of TSS and CpG islands within 1-kb of the transcripts are shown in the inserted histograms. Annotated genes and randomly selected intergenic sites are also shown as positive and negative controls, respectively. (**D**) On the basis of population genetics data in rhesus macaque, the distribution of π for synonymous sites (***Syn Sites***), non-synonymous sites (***Nonsyn Sites***), all lncRNAs, lncRNA precursors and non-coding genes (***Functional***) are summarized in boxplots. *NS*: not significant, **p-value <0.01.

We first compiled the whole lncRNAome in rhesus macaque using a similar strategy as described previously [31], on the basis of strand-specific RNA-Seq in ten tissues from the same macaque animal [16,32] (**Fig 2A; Materials and Methods**). A total of 5,641 lncRNA transcripts were assembled, verifying known features of transcripts such as the tight association with epigenetic markers and CpG islands [33,34] (**Figs 2B and 2C** and **S3**). Moreover, as positive control, we compiled a list of 89 non-coding genes in rhesus macaque. These non-coding genes are reportedly functional in human, as supported by experimental evidence [35]. Given the existence of the similar lncRNA transcripts in rhesus macaque, we assumed that these macaque lncRNA transcripts may also have functions and are under similar selective constraints as annotated non-coding genes in human (**Materials and Methods**).

To quantify the level of selective constraints, we then performed whole genome sequencing of 24 independent macaque animals and generated 23.7 billion paired-end reads with high quality, yielding high sequencing coverage of the macaque genome (ranging from 26- to 70-fold). On the basis of these sequencing data, as well as seven public datasets for macaque genomes [36–38], we profiled 54,079,575 single nucleotide polymorphic sites across the macaque genome, yielding a considerable number of polymorphisms located on the lncRNA loci (**S4 Table; Materials and Methods**).

On the basis of the polymorphism data in the population of 31 unrelated macaque animals, we measured the level of polymorphisms in the subset of macaque lncRNA loci, whose orthologous regions in human were *de novo* genes, and compared that to the same measures in all macaque lncRNAs, as well as the list of 89 established non-coding genes as a reference [35] (**Materials and Methods**).

As expected, we found that the list of 89 established non-coding genes are selectively constrained in rhesus macaque on the basis of the significantly decreased nucleotide diversity (π), compared with the synonymous sites of known macaque coding genes as a neutral control (*Wilcoxon one tail test*, *p-value* = 0.008, **Fig 2D**). The selection on these non-coding genes seems to be moderate, as compared with that of the non-synonymous sites of known macaque coding genes as a benchmark (**Fig 2D**). In contrast to this small repertoire of non-coding genes with functions, it seems that lncRNA transcripts are in general not selectively constrained in rhesus macaque, with the nucleotide diversity comparable with that of the synonymous sites across the macaque genome as a neutral control (*Wilcoxon test*, *p-value* = 0.917, **Fig 2D**). In addition, the orthologous loci for the lncRNA precursors of human *de novo* genes are not subjected to strong selective constraints as those non-coding genes, with the population genetics feature indistinguishable from that of the synonymous sites (*Wilcoxon test*, *p-value* = 0.570; **Fig 2D**), as well as that of the whole lncRNA pool (*Wilcoxon test*, *p-value* = 0.449; **Fig 2D; Materials and Methods**). In conclusion, we didn't find evidence for higher selective constraints for the orthologous lncRNA loci for human *de novo* gene precursors. Hence, it seems likely that the ancestor of *de novo* genes may not be particularly distinct in terms of functional importance before the proteins arise.

### The existence of *de novo* proteins is not beyond anticipation in terms of their theoretical lifespan

Given that the lncRNA precursors for human *de novo* genes did not display particularly distinct functional status, it is interesting to investigate whether other features, such as sequence features, may explain why *de novo* genes originate from some lncRNAs but not the others. In addition, given the smaller effective population size in hominoids, the detection of *de novo* proteins might arise from translational noise that is not acted upon or not yet removed by purifying selection, rather than being positively selected for due to their newly-acquired protein-level functions. We thus performed comprehensive sequence analysis of these *de novo* genes to investigate whether any sequence features could underlie the biased origination process of *de novo* genes from a subset of lncRNAs, and whether the existence of these *de novo* proteins is beyond anticipation in terms of their theoretical lifespan.

**Fig 3:**
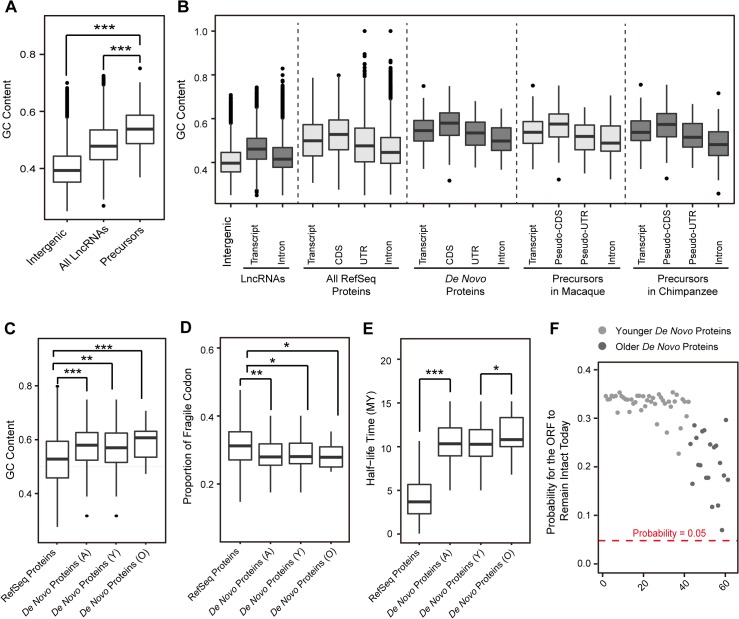
Emergence of human *de novo* proteins from GC-rich lncRNA precursors. **(A)** GC contents for randomly-selected intergenic regions, all lncRNAs and lncRNA precursors in rhesus macaque are summarized in boxplots. (**B**) GC contents of different genomic regions are shown for *de novo* genes in human, as well as the orthologous non-coding regions in chimpanzee and rhesus macaque. For lncRNA precursors, the *pseudo*-CDS and *pseudo*-UTR regions were defined according to the orthologous relationship with the corresponding CDS and UTR regions of human *de novo* proteins. (**C**) GC contents for CDS regions of RefSeq proteins and *de novo* proteins in human are summarized in boxplots. A: all *de novo* genes, Y: younger *de novo* genes, O: older *de novo* genes. **(D)** Boxplot showing the distribution of fragile codon composition of *de novo* genes and RefSeq proteins in human. **(E)** Boxplot showing the distribution of half-life time of *de novo* genes and RefSeq proteins in human. (**F**) Dot plot showing the survival probability of the *de novo* ORFs. The probability of 0.05 was marked by red dashed line.

Interestingly, when analyzing the sequence features of these orthologous lncRNA precursors, we found that they have significantly higher GC contents in comparison with other lncRNAs and non-genic regions (**Fig 3A** and **3B**; *Wilcoxon one-tailed tests*, *p-value*<1.0e-7 for both comparisons). The ORFs of *de novo* genes derived from GC-rich lncRNAs were also observed to have significantly higher GC content when compared with functional proteins in RefSeq (**Fig 3B** and **3C**; Median of GC content = 0.57 *vs*. 0.53; *Wilcoxon* one-tailed test, *p-value* = 6.6e-6).

Further investigation revealed that such a GC-rich property endows these newly-originated ORFs with longer theoretical lifespan, even longer than their current age. As the stop codons are AT-rich, the higher GC content usually supports relatively stable ORF compared with GC-poor sequences [39,40] (**S4 Fig**). As expected, compared with RefSeq proteins, these newly originated ORFs have less content of fragile codons–codons convertible to stop codon by a single point-mutation, and are thus less susceptible to non-sense mutations (**Fig 3D**; *Wilcoxon* one-tailed test, *p-value* = 0.002). Accordingly, we found that these ORFs have long half-life time under neutrality (**Materials and Methods**), even significantly longer than other functional proteins in RefSeq (**Fig 3E**; *Wilcoxon* one-tailed test, *p-value*<2.2e-16). Especially, compared with the younger *de novo* genes, the older *de novo* genes have higher GC content (**Fig 3C**), less content of fragile codons (**Fig 3D**) and longer half-life time (**Fig 3E**). Overall, the theoretical lifespan of these newly-originated proteins is generally longer than their current age (**Figs 1B** and **3E**), thus indicating that the existence of these *de novo* proteins is not beyond anticipation even under neutral expectation (**Fig 3F)**.

Overall, the *de novo* gene repertoire we identified in the hominoid lineage actually represents a snapshot for the steady-state representation of a dynamic turnover process of ORFs. The detection of these GC-rich *de novo* proteins with stable ORFs, together with the previous reports that many *de novo* genes have stable expression profiles possibly by sharing the transcriptional context with nearby protein-coding genes through *cis*-natural antisense or bi-directional promoters [16,41], seems to favor the notion that a significant portion of the turnover is probably driven by genetic drift and those GC-rich "survivors" with long ORF lifespan and stable expression profile were retained and detected during a birth-and-death process.

### The new *de novo* proteins are under selective constraints in human population

What we found above suggest that the emergence and retention of *de novo* genes are likely under neutral forces. However, considering these GC-rich “survivors” have been exposed to natural selection for relatively long time due to their theoretically longer lifespan, it is interesting to investigate whether some of these newly originated ORFs have been maintained by selective constraint in the current population, due to their newly-acquired protein-level functions. We thus performed population genetics study in human and rhesus macaque populations to assess whether selective constraints are applied to these ORF regions in human populations but not their non-coding counterparts in rhesus macaque.

**Fig 4:**
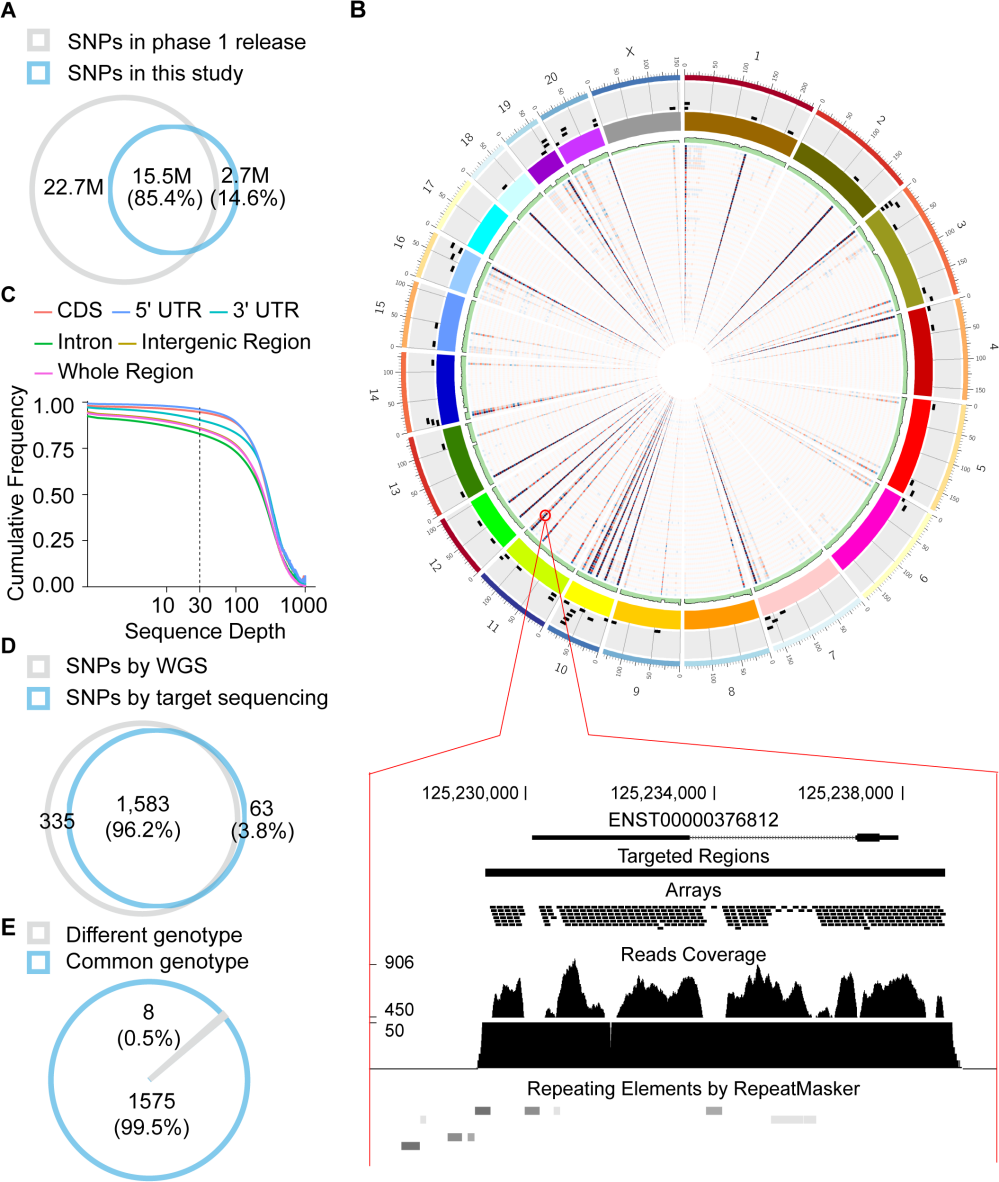
Profiling of polymorphisms in human and rhesus macaque. **(A)** Comparison of human polymorphism sites profiled in this study with those in the 1000 Genomes Project. (**B**) The sequencing coverages of whole genome sequencing from one macaque animal and for the targeted re-sequencing of 82 macaque animals are summarized in green barplot and heatmaps inside the *Circos* map, respectively. The depths of the sequencing coverage are proportional to the color depth. Black rectangles outside the colored chromosome block represent the genomic locations of macaque orthologous regions of human *de novo* genes. The bottom panel illustrates the sequencing details of one region of interest. (**C**) Cumulative frequency of mean sequencing coverage on different genic regions of *de novo* genes is shown. Intergenic regions: 1-kb regions upstream and downstream of the gene. (**D, E**) Venn diagrams showing the distributions of macaque polymorphism sites identified by whole-genome sequencing and targeted re-sequencing, in terms of polymorphism sites (**D**) and genotypes (**E**).

**Fig 5:**
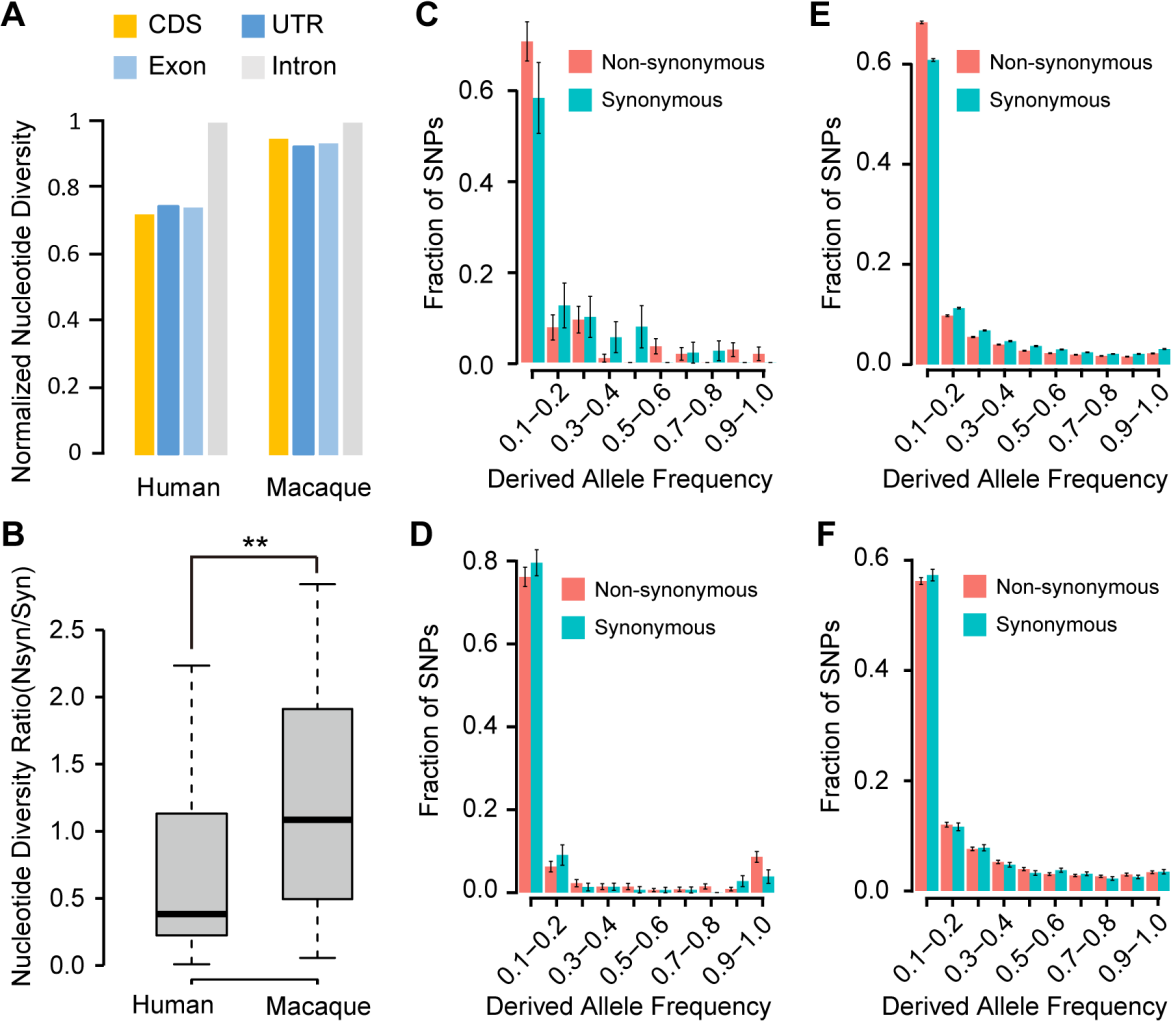
Evidence of purifying selection on the human *de novo* genes. **(A)** Comparison of π in different genomic regions. The values were normalized with that of intronic regions. **(B)** The ratios of π for non-synonymous sites to synonymous sites for *de novo* genes or orthologs in rhesus macaque were summarized in boxplots (**C-F**) Derived allele spectra for *de novo* genes (**C**), protein-coding genes (**E**) and lncRNAs (**F**) in human, as well as for the macaque regions orthologous to the human *de novo* genes (**D**) are shown. The standard deviations estimated by 1,000 bootstrap replicates are indicated by the error bars.

We first profiled a set of polymorphism sites in human populations, by re-analyzing whole genome sequencing data in 67 individuals from different sub-populations (**Fig 4A** and **S5 Table; Materials and Methods**). We expect that if the *de novo* genes encode functional proteins and are maintained by purifying selection, the polymorphism level for exonic regions of these genes will be lower than intronic regions. The polymorphism level for non-synonymous sites should also be significantly lower than that of synonymous sites, as the former will be under much stronger selection. Moreover, we expect to find a difference in the frequency spectra at nonsynonymous *vs*. synonymous sites, resulting in a skew towards low frequency variants compared to the latter. These are indeed what we found: 1) The *θ*_*w*_ and π measures were significantly lower in the exonic region of the *de novo* genes compared to the intronic region of the same locus (*Monte Carlo p-values*<1e-4; **Figs 5A** and **S5**; **Materials and Methods**). In addition, the UTR regions of these *de novo* genes showed *θ*_*w*_ and π measures that are lower than the intronic regions, while slightly higher than CDS regions (**Figs 5A** and **S5**). 2) Compared with synonymous sites, the nucleotide diversity for non-synonymous sites was significantly lower (*Wilcoxon one-tail test*, *p-value* = 0.019; **S6 Fig**). Accordingly, the ratio of the nucleotide diversity for non-synonymous sites to synonymous sites was generally smaller than 1 (**Fig 5B**). 3) The frequency spectrum of the derived alleles had an excess of low-frequency variants at the non-synonymous sites in the *de novo* genes compared to that at the synonymous sites (**Fig 5C**), which is similar to known protein-coding genes (**Fig 5E**). As a control, we classified mutations in human lncRNAs into synonymous or non-synonymous sites within the longest *pseudo*-ORFs, and didn’t observe any difference in their respective frequency spectrum (**Fig 5F; Materials and Methods**).

Accordingly, as a negative control, we performed population genetics study on macaque orthologous regions (without coding potential) of human *de novo* genes, in a population of 82 unrelated rhesus macaque animals (**Materials and Methods**). Custom library with >135,000 120-bp DNA oligos were designed to capture the macaque orthologous regions. Ultra-deep sequencing was then performed (**Materials and Methods**) and 222 million 150-bp paired-end reads were generated and uniquely located on the macaque genome (NCBI SRA accession number: SRP052932; **S6 Table**). The average effective coverage of the targeting regions reached to 94% in each sample (**Figs 4B and 4C** and **S7**), and a total of 10,162 single nucleotide polymorphisms were identified across the target genomic regions with high sensitivity and specificity, as verified by a follow-up whole genome sequencing with 30× coverage in one of these macaque animals (**Fig 4D** and **4E**; **Materials and Methods**).

On the basis of the polymorphism data of the macaque orthologous regions, we found that both the *θ*_*w*_ and π measures were uniform across the length of these regions, in contrast to the clear differences observed in human (**Figs 5A** and **S5**). We further classified the polymorphism sites on macaque lncRNAs into synonymous or non-synonymous sites, according to codon-level alignments between human *de novo* proteins and their orthologous lncRNAs in rhesus macaque. No significant difference was detected for the nucleotide diversity of *pseudo*-non-synonymous and *pseudo*-synonymous sites in rhesus macaque (*Wilcoxon one-tail test*, *p-value* = 0.607; **S6 Fig**), with the ratio of the nucleotide diversity between these two groups comparable to 1 (**Fig 5B**). In addition, the resulting frequency spectrum of derived alleles at the *pseudo*-non-synonymous sites is indistinguishable from that at the *pseudo*-synonymous sites (**Fig 5D**). The population genetics analyses thus suggest that these newly-originated *de novo* genes have gained new functions specifically in human.

Taken together, although the *de novo* proteins seem to emerge from lncRNA precursors with no bias towards those functionally-constrained lncRNAs, and their existence is not beyond the anticipation under neutral expectation, at least a proportion of these proteins should have acquired protein-level functions specifically in human, as revealed by the species-specific signatures of purifying selection on these newly-originated *de novo* genes. We thus depicted a new mechanism for the origination of functional proteins from ancestral non-coding transcripts with precise splice structures and specific tissue expression profiles during the primate evolution.

## Discussion

### *De novo* origination of protein-coding genes could be more pervasive in primates

Although our current study identified a list of 64 hominoid-specific *de novo* genes, more proteins are expected to originate through this *de novo* mechanism [42]. Several types of *de novo* genes might be underrepresented due to the computational pipelines currently used to identify these genes: 1) Genes with shorter ORF. Automatic gene annotation pipeline typically neglect genes with short ORFs by using arbitrary criteria to define the minimal ORF length. Consequently, considering *de novo* proteins generally have short ORFs [43–45], a large proportion of *de novo* proteins with short ORFs were removed. 2) Genes without orthologous regions in out-group species were not included, due to the requirements of the high-quality alignments for accurate age assignments of ORFs in vertebrate phylogeny, as well as of the detection of common ancestral “disablers” as indication for newly-created rather than old dying genes. Although such a design effectively lowered the false-positives and potential bias introduced by blast-like alignments in *de novo* gene identification [27], some false-negatives could still result from our stringent criteria. 3) Genes with non-stable accession numbers. Considering the difficulties in defining the coding potential, the Ensembl accession numbers assigned to *de novo* genes are typically not stable. Although our study has combined multiple versions of Ensembl databases to identify *de novo* genes, some genes may still be overlooked. 4) Genes with low expression. *De novo* genes typically express in low abundance [13,16,18]. As reliable evidence for transcriptional and translational expression is needed to define a *de novo* gene, especially considering the relatively low sensitivity of mass spectrometry technology to reliably detect peptides of low abundance, these genes may be missed. 5) Additionally, the current identification pipeline by comparative genomics approaches typically focuses on in-group ORFs that are missed in other out-group species. In such an occasion, a considerable proportion of *de novo* genes originated through lineage-specific expression of pre-existing ORFs might be neglected [17,18].

Overall, although these 64 genes may not fully recapitulate the true repertoire of *de novo* genes in the hominoid lineage, they should constitute a representative group for further analysis and elucidation of the evolving process of *de novo* genes from precursor lncRNAs.

### Origination of functional proteins from ancestral lncRNAs

Although *de novo* genes are also regarded as “motherless” genes due to their lack of ancestral protein-coding genes as precursors, we and others have found that at least a proportion of lncRNAs might represent an intermediate stage of their origination, narrowing the gap between non-coding DNA and protein-coding genes. Such an origination process may take advantage of existed genomic contexts. For example, these lncRNA “precursors” usually share the transcriptional context with the nearby protein-coding genes through *cis*-natural antisense or bi-directional promoters [16,41]. These lncRNAs with stable expression profiles, although not more selectively constrained according to our population genetics study, may then lay the foundation for the emergence of new *de novo* genes. In addition, the GC-rich sequence property of these lncRNAs further supports stable ORFs of the newly-originated proteins (**Fig 3**). Overall, these genomic features provide a theoretically favorable foundation for the birth of some functional proteins–a notion well supported by our population genetics data, which revealed that some of these loci already encode human-specific functional proteins (**Fig 5**).

Although such an origination process is plausible, currently both the definitions of *de novo* genes and lncRNAs are depending on some arbitrary criteria [9,13,31]. Additional lines of evidence are thus needed to fully support the mechanism through which *de novo* genes come from the lncRNA pools during the primate evolution. For example, since it is still technically challenging to fully annotate proteomes based on mass-spec studies across tissues, development stages and species, it is inadequate to directly identify human *de novo* genes on the basis of the presence or absence of peptides across different species. Alternatively, conceptual translation of ORFs between species is still the main strategy in the field to infer the existence of these *de novo* proteins in different out-group species [9,13]. In this context, although the orthologs of these human *de novo* genes could be defined as “lncRNAs” in chimpanzee and rhesus macaque by the current criteria, they may actually encode smaller version of these *de novo* proteins in out-group species.

We thus performed cross-species analyses to test this “functional ORF expansion model”. Briefly, if the functional proteins were indeed absent from out-group species, we would expect similar substitution rates between non-synonymous and synonymous sites when performing comparative genomics analysis. When aligning the truncated forms of the human *de novo* proteins in non-human primates, we found that the merged dN/dS ratio does not deviate significantly from 1 (dN/dS = 0.90). In line with this finding, our population genetics study on macaque orthologous regions of human *de novo* genes also indicated that these macaque orthologs may not encode similar functional proteins as in human (**Fig 5**). Even considering these population genetics evidence, we still could not fully exclude the possibility that some of these so-called “lncRNAs” might actually encode fast-evolving or smaller version of the protein in out-group species. As such, these proteins might be under weak selection, and the signals for selective constraints could not be detected based on the population size of this study. Future mass-spec studies with high sensitivity may aid in clarifying these issues.

### “GC-rich inheritance model”: an alternative explanation to the GC-rich feature of coding regions

Protein-coding genes have typically higher GC content than non-coding regions. It has been proposed that the increased GC content in genic regions could be maintained by natural selection, such as the GC preference on the wobble sites potentially shaped by adaptive evolution for the stability of mRNA secondary structure or the efficient protein synthesis [46].

However, here we found that other gene-associated genomic regions are also GC-rich, such as the intronic regions (**Fig 3B**). Theoretically, considering the models for new gene origination, each protein-coding gene could be traced to an ancient origination event from non-coding DNA. There is thus a formal but as yet unexplored possibility that the biased inheritance from GC-rich lncRNAs could be another major factor underpinning the different extents of GC content between coding regions and genomic background. As we provided an evolutionary and functional connection between protein-coding genes and non-coding DNA regions in the hominoid lineage, we formally tested this “GC-rich inheritance model”. Correspondingly, we found that GC-rich lncRNAs are favorable precursors for new proteins. More importantly, the GC-rich features could be detected in all genomic regions associated with these newly originated *de novo* genes, even for the wobble sites with established GC preference, as well as their lncRNA precursors, which also resembled those of the well-known protein-coding genes (**Figs 3B** and **S8**). Besides being a consequence of adaptive evolution after the acquirements of the ORFs, the GC-rich feature of the protein-coding genes may also inherit from the ancestor lncRNAs, thus complementing previous theory on GC-rich feature for protein-coding genes [46–49].

## Materials and Methods

### Ethics statement

Rhesus macaque samples were obtained and manipulated from the internationally-accredited (Association for Assessment and Accreditation of Laboratory Animal Care, AAALAC) animal facility of the Institute of Molecular Medicine in Peking University. The present study was approved by the Institutional Animal Care and Use Committee of Peking University.

### Identification and characteristics of *de novo* protein-coding genes in the hominoid lineage

*De novo* protein-coding genes in the hominoid lineage were identified using a genome-wide pipeline integrating *ab initio* identifications and meta-analysis of public datasets. On the basis of Ensembl gene annotations (v68), *de novo* genes were identified using a similar pipeline as we published previously [16]. Briefly, 1) we inferred the locus ages on the basis of the syntenic genomic alignment generated by UCSC, and only human genes assigned with specific locus age were retained; 2) for locus with high-quality alignment (coverage >70% and identity >50%) in the out-group species, the existence of the ORF in multiple out-groups (chimpanzee, orangutan, rhesus macaque, mouse, guinea pig, dog, hedgehog and armadillo) was inferred separately by Exonerate [50], and if the sequence in particular out-group encoded at least one frame-disrupting indel or premature stop codon, with the subsequent maximum continuous ORF shorter than 70% of the human ORF length (a cutoff based on the previous practice in this field [9,13,16]), the ORF was regarded as non-existent in this out-group; 3) we inferred the origination timing of ORFs for these *de novo* genes by summing up information of their presence or absence in multiple out-group species, along with the phylogenetic tree with the principle of parsimony, and subsequently retained only genes originated in the hominoid lineage; 4) sequence alignments were then performed against all human proteins (BLAST *e-value* cutoff of 10^−6^) to ensure these new genes originated through *de novo* evolution other than gene duplications. Finally, 56 protein-coding genes were identified as candidate *de novo* genes in the hominoid lineage.

The resulted 56 candidate genes, together with 99 literature-documenting primate-specific *de novo* genes [9–11,13,16], were then subject to two additional inclusion criteria. First, genome-wide expression filters were introduced to ensure these genes had convincing evidence for transcriptional and translational expression in human. Public RNA-Seq data in 17 human tissues (Human BodyMap 2.0 data from Illumina and data from references [51,52]) were integrated and analyzed to estimate the gene expression level of each gene, according to a standardized pipeline [53]. To distinguish true transcription signals from the background expression, we first estimated the RPKM values for the genomic background represented by 10,000 randomly-selected intergenic regions. The expression levels of intergenic regions were significantly lower than 0.2 RPKM in all tissues (**S1A Fig;** *Monte Carlo p-values* ranging from 0.002 to 0.028). Therefore, a more conservative PRKM cutoff of 0.5 was arbitrarily set to confirm the transcriptional expression of these *de novo* genes in human. Two candidates (ENSG00000205056 and ENSG00000198547) with low RPKM scores were also included due to their reliable experimental evidence for transcriptional expression [9,11]. Peptide evidences from large-scale mass spectrometry studies were then extracted from PRIDE [28], PeptideAtlas [54], ProteomicsDB [55] and Human Proteome Map [29]. A peptide was considered to support the protein expression of a *de novo* gene only if 1) when performing BLAT similarity searches against all human proteins (Ensembl v68, BLAT settings-*t = prot-q = prot-stepSize = 5*), its whole sequence exactly match the CDS region of the *de novo* gene, with the second-best hit in the proteome (if existing) including at least one mismatch; and 2) when performing BLAT similarity searches against the human genome, its whole sequence identically and exclusively match the CDS region of the *de novo* gene (hg19, BLAT settings-*stepSize = 5-stepSize = 5-t = dnax-q = prot*). Only genes with 1) RNA-Seq RPKM >0.5 in at least one of the 17 human tissues, and 2) at least one convincing item of peptide evidence in support, were retained (**Fig 1A**). Second, to verify that these genes are newly-originated rather than old dying genes, we manually checked the corresponding ORF regions in multiple out-group species (chimpanzee, orangutan, rhesus macaque, mouse, guinea pig, dog, hedgehog and armadillo), and only genes with common ancestral disablers shared by multiple out-group species were retained (**Fig 1A**). Here, a common ancestral disabler refers to a mutation disrupting the ORF in multiple out-group species at the same sequence position [9,11]. In such scenario, the mutation is more likely to be of an ancestral status according to the parsimony principle, thus indicating the gene is newly-originated rather than old dying. Totally, a list of 64 genes was identified to originate recently in the hominoid lineage through *de novo* evolution (**Fig 1B** and **Tables 1, S1 and S2**).

We also studied the characteristics of these *de novo* genes across primate species in the context of new genomics technologies. According to computational pipelines described previously [9,13,16], mRNA and EST data from UCSC Genome Browser, RNA-Seq data archived in RhesusBase [53,56], as well as single-molecule long-read sequencing data on human transcriptome [57] were downloaded and analyzed to investigate the transcriptional structure of these *de novo* genes in human. On the basis of public RNA-Seq data in human, chimpanzee and rhesus macaque [16,51,52], comparative transcriptome studies were then performed to compare the transcription level, splicing structure and tissue expression profiles of these *de novo* genes with their non-coding orthologs in chimpanzee and rhesus macaque, according to a pipeline previously described by us [16]. Specially, an RPKM cutoff of 0.2 was set to distinguish convincing transcription and transcriptional noise as described above (**S1B** and **S1C Fig**).

### LncRNA identifications in rhesus macaque

Strand-specific, Poly(A)-positive RNA-Seq data in ten tissues (adipose, prefrontal cortex, cerebellum, heart, kidney, liver, lung, muscle, spleen, testis) of the same macaque animal were used to assemble the lncRNAome in rhesus macaque [16,32], following a computational pipeline as described previously [31]. Briefly, RNA-Seq reads of each macaque tissue were aligned separately to the macaque genome (rheMac2) with Tophat (v2.0.6) [58]. Transcriptome assembly was then performed with both Cufflinks (v2.0.2) and Scripture (VPaperR3) [59,60], and redundant transcripts were merged with Cuffcompare (v2.0.2) [60] after boundary correction. To control for false-positives, only long, multi-exonic transcripts (>200 bp) with supportive evidences in ≥2 tissues or by both assemblers were retained [31,61]. To evaluate the performance of this transcriptome assembly, Cuffcompare (v2.0.2) was also introduced to compare the assembled transcripts with multi-exonic protein-coding genes as annotated in RefSeq. Finally, a total of 90,322 multi-exonic transcripts were assembled, which represents transcript structures reconstructing for 95% known multi-exonic protein-coding genes, suggesting the feasibility of this assembly strategy (**S3 Fig**).

Several stringent criteria, such as proteome annotation- and comparative genomics-based filtering procedures, were incorporated to exclude protein-coding transcripts. Briefly, 1) transcripts with ≥1 splice junction overlapped with known protein-coding genes annotated in either Ensembl or RefSeq were discarded; 2) PhyloCSF was applied to score the coding potential of these candidates (multiple sequence alignments of 9 mammalian genomes,—*frames = 3—orf = StopStop3*) [62] and only transcripts with PhyloCSFscore <65 were retained, corresponding to a false negative rate of 1% and a false positive rate of 5% on the basis of RefSeq annotation. 3) the nucleotide sequences or 3-frame stop-to-stop translation products were subjected to Blastx, Blastp and HMMER searching against all human proteins or known protein domains (Pfam-A, Pfam-B) [63], and transcripts with significant hits (*e-value* ≤10^−4^) were discarded; 4) transcripts with putative ORFs ≥100aa longer were also discarded. The strategy had a good performance in distinguishing lncRNAs from protein-coding transcripts (**S3 Fig**) and a total of 5,641 lncRNA transcripts were assembled. On the other hand, non-coding genes in human were searched and downloaded from lncRNAdb (http://www.lncrnadb.org/) [35] and the macaque orthologs of these functional human lncRNAs were then retrieved by liftOver.

### Characterization of sequence features

For human *de novo* genes and their orthologs in chimpanzee and rhesus macaque, sequences of different genomic regions were retrieved and the GC contents were calculated by a customized Perl script (https://github.com/Jia-Yu-Chen). Following a previous study [40], we also calculated for each *de novo* gene the proportion of fragile codons that could become stop codons by single mutation. We further investigated whether the existence of these *de novo* proteins is beyond anticipation in terms of their theoretical lifespan. Briefly, an ORF would eventually be interrupted if it does not experience any functional constraint, and the rate of ORF interruption under neutrality is largely determined by point mutation rate and insertion/deletion mutation rate. We thus estimated the interruption rate in terms of half-life time of ORF (t_1/2_) according to the computational simulation method developed by Zhang and Webb [64] (**Fig 3E**). The half-life time of a given ORF is the time required for an ORF to be interrupted in one-half of 20,000 simulation replicates, with the rates of point and insertion/deletion mutations being set as 1.25 and 0.1 per site per billion years, respectively, as previously defined [64]. Given this half-life time (*t*_*1/2*_), the minimal probability of an ORF remaining intact today under neutrality was then determined by the following equation, by assuming that the lineage-specific gene emerged right after the species divergence from the most recent common ancestor (*T*) (**Fig 3F**).

$$p={\lambda e}^{-\lambda },\;where\;\lambda ={\left(1/2\right)}^{T/t_{1/2}}$$

### Targeted and whole genome sequencing and polymorphism identification

We firstly profiled the polymorphism data in human populations, by re-analyzing whole genome sequencing data in 67 individuals from different sub-populations and archived with high sequencing coverage by the 1000 Genomes Project (**S5 Table**). Briefly, deep sequencing reads were mapped to the human genome (hg19) using BWA [65], and the polymorphism sites of each sample were identified and evaluated according to the standard GATK pipeline with UnifiedGenotyper (V2.7–4) [66]. After stringent filtering strategies to remove false-positives in variant calling, 18,186,523 highly reliable single nucleotide variants were identified across the human genome, with 85.4% supported also by the 1000 Genomes Project (**Fig 4A**).

Accordingly, we profiled the distributions of polymorphic sites in rhesus macaque populations as a reference. For each human *de novo* gene, we performed targeted capture and ultra-deep sequencing of the macaque orthologous regions (and 1kb flanking regions) in a population of 82 unrelated male animals (**S6 Table**). Briefly, custom library with >135,000 120-bp DNA oligo probes were designed by Agilent SureSelect XT Target Enrichment System (Agilent Technologies, Inc., Santa Clara, USA), with a 3-folds tiling coverage, to capture the targeted regions in rhesus macaque. Genomic DNA from the macaque animals was isolated from 200–500*μl* whole blood using the QIAamp DNA Blood Mini Kit (Qiagen, Venlo, Netherlands) and 3*μg* DNA of each animal was sheared to fragments with a peak at 150–200bp using Covaris S220. Then, the adaptor-ligated libraries were amplified, purified and hybridized with SureSelect Capture Library according to the manufacturer’s instructions. After 16h hybridization at 65°C, the captured targets were pulled down by Dynabeads MyOne Streptavidine T1 (Life Technologies, Ltd., Carlsbad, USA) and amplified for the library preparation, which were then sequenced on Illumina Miseq system with 151-bp paired-end read mode. Totally 222 million 150-bp paired-end reads were generated and uniquely located on the macaque genome (**S6 Table**). The average effective coverage of the targeted regions reached to 94% in each sample (**Fig 4B**), and 86% of whole orthologous region (or 95% of CDS regions) were sequenced with coverage of ≥30 in all of the 82 macaque animals (**Figs 4C** and **S6**). A total of 10,162 highly-reliable single nucleotide polymorphisms were then identified, according to the standard GATK pipeline with UnifiedGenotyper (V2.7–4) [66].

To evaluate whether allele dropout or other false-positives introduced by target capture may compromise our approach, we further performed whole genome sequencing with 30× coverage in one of these macaque animals (Animal ID: 920653) for evaluation (**Fig 4B**). Genomic DNA was obtained for the library preparation of whole genome re-sequencing, deep sequencing was performed on a HiSeq 2000 Sequencing System with a 151×2 paired-end read mode, and single nucleotide polymorphisms were identified according to the standard GATK pipeline. Finally, 96.2% polymorphic sites identified in the targeted sequencing were verified by the whole genome sequencing, with 99.5% showing the same genotype (**Fig 4D** and **4E**).

Whole genome sequencing data from 24 macaque animals generated previously in our lab, as well as seven animals published previously [36–38], were also analyzed to profile a genome-wide polymorphism dataset across the macaque genome, according to the pipeline as described above. All deep sequencing data in this study are available at NCBI SRA under accession numbers SRP052932.

### Population genetic analyses in human and rhesus macaque

On the basis of the polymorphism data from the population of 31 macaque animals, we measured the nucleotide diversity (π) for the orthologous lncRNA loci of human *de novo* genes, all lncRNAs, and a list of 89 functional non-coding genes in rhesus macaque. Non-synonymous and synonymous sites of macaque protein-coding genes as annotated by RefSeq were used as benchmarks for the extent of the selective constraints (**Fig 2D**). *Wilcoxon* test was performed to test whether the nucleotide diversity between two groups are significantly different, with a *p-value* cutoff of 0.05 (**Fig 2D**).

On the basis of the polymorphism data obtained by analyzing the whole genome sequencing data of 67 human individuals and the targeted sequencing data of 82 macaque animals, we estimated the polymorphism levels (*θ*_*w*_ and π) for different genomic regions (exon, intron, CDS and UTR) of the *de novo* genes in human and their orthologs in rhesus macaque (**Figs 5A** and **S5**). We further performed statistical tests to determine whether the different polymorphism levels between exonic and intronic regions of human *de novo* genes are statistically significant, with the background estimated by 10,000 times of *Monte Carlo* simulations, assuming the polymorphic sites were randomly distributed in exonic and intronic regions of human *de novo* genes. Considering that the average nucleotide diversity in rhesus macaque is higher [67], if the exonic regions are more selectively constrained than intronic regions, we should have greater statistical power to detect the difference. The observation of a comparable nucleotide diversity between macaque exonic and intronic regions then indicates that these macaque orthologs of human *de novo* genes may not encode similar functional proteins as in human (**Figs 5A** and **S5**).

The ratio of the nucleotide diversity between non-synonymous sites to synonymous sites was also determined for each *de novo* gene, as well as its non-coding ortholog in rhesus macaque (**Fig 5B**), in which the *pseudo*-non-synonymous and *pseudo*-synonymous sites in macaque orthologs were determined by codon-level alignment with human *de novo* proteins. For each polymorphic site, the derived allele was defined by the EPO pipeline [68,69]. The frequency spectra of derived alleles were then estimated, with 1,000 times of bootstrap performed to estimate the confidence intervals of the proportions of polymorphism sites (**Fig 5C–5F**). Similar analyses were performed for known protein-coding genes as annotated by RefSeq, as well as human lncRNAs as annotated by GENCODE (v19) as controls.

## Supporting Information

S1 Fig Estimation of background expression levels represented by RPKM values of intergenic regions. The RPKM values of 10,000 randomly-selected intergenic regions were calculated for tissue samples from human (**A**), chimpanzee (**B**) and rhesus macaque (**C**). The distribution of RPKM values was shown and the percentages of regions with RPKM>0.2 was calculated to estimate the *p-values* for genomic background transcription with an RPKM cutoff of 0.2.
(TIF)

S2 Fig Hierarchical clustering of tissue expression profile. For human *de novo* genes and their orthologs in chimpanzee and rhesus macaque, the expression levels in different tissues were calculated in terms of RPKM. The RPKM values were then clustered according to similarity using complete linkage hierarchical clustering. Grey boxes: missing data.
(TIF)

S3 Fig Evaluations on pipeline for macaque lncRNAome identification. (**A**) Pie chart showing the percentage of multi-exonic protein-coding transcripts annotated by RefSeq, and reconstructed by our pipeline. The percentages of full overlap, partial overlap and junction support transcripts were calculated and shown. (**B**) Using *PhyloCSF* score of 65 as the threshold, the percentage of protein-coding genes and non-coding genes annotated by RefSeq below or above the threshold were shown, respectively.
(TIF)

S4 Fig Correlation of GC content with the fragile codons. The percentages of fragile codons are plotted against the GC content for known protein-coding genes as annotated by RefSeq.
(TIF)

S5 Fig Comparison of *θ*_w_ in different genomic regions. The *θ*_*w*_ values were calculated for different regions and then normalized with that of intronic regions.
(TIF)

S6 Fig Distributions of nucleotide diversity for human *de novo* genes and their macaque orthologs. The distributions of nucleotide diversity were summarized as boxplots for human (**A**) and rhesus macaque (**B**), respectively. The *pseudo*-non-synonymous and *pseudo*-synonymous sites in macaque orthologs were determined by codon-level alignment with human *de novo* proteins.
(JPG)

S7 Fig Cumulative frequency distribution of sequencing coverage for 82 macaque samples. For each macaque sample, cumulative frequency distributions of sequencing coverage for different genomic regions are plotted separately.
(TIF)

S8 Fig GC contents at the third codons. The GC contents at the third codons for *de novo* genes and known protein-coding genes as annotated by RefSeq are summarized in boxplots, respectively.
(TIF)

S1 Table Annotations for 64 *de novo* protein-coding genes. (XLSX)

S2 Table Evidence supporting the transcription and translation of *de novo* protein-coding genes in human. (PDF)

S3 Table Expression levels of human *de novo* genes and their orthologs in chimpanzee and rhesus macaque. (XLSX)

S4 Table Statistics of polymorphic sites in rhesus macaque. (PDF)

S5 Table Information of 67 human individuals with whole genome re-sequencing data. (PDF)

S6 Table Statistics of targeted sequencing in 82 macaque animals. (PDF)
