# Supplementary figures and images for: Emergence, Retention and Selection: A Trilogy of Origination for Functional De Novo Proteins from Ancestral LncRNAs in Primates
Source: PLoS Genet. 2015 Jul 15;11(7):e1005391. doi: 10.1371/journal.pgen.1005391 (PMC4503675; doi:10.1371/journal.pgen.1005391)

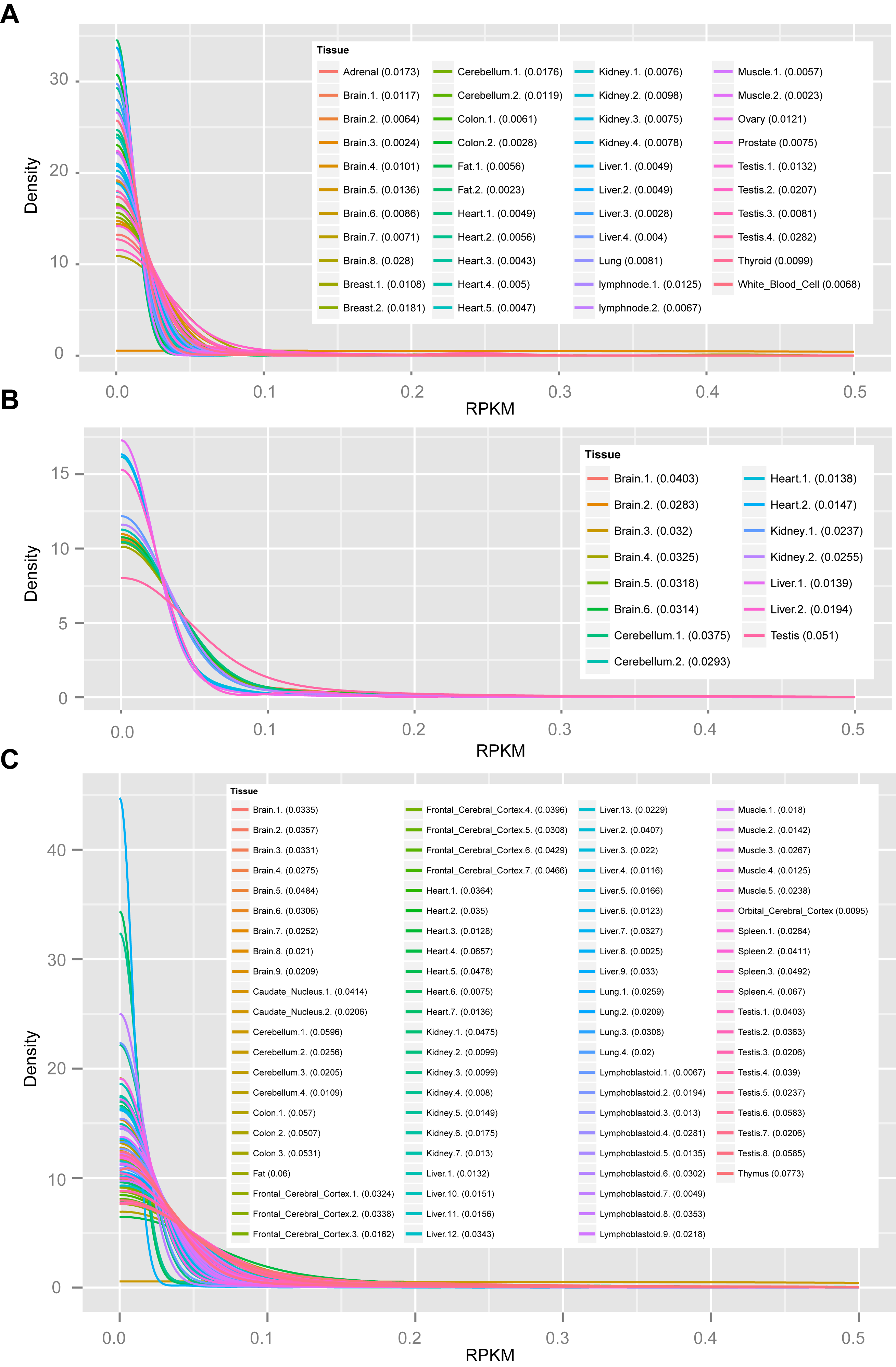

Supplement: S1 Fig — The RPKM values of 10,000 randomly-selected intergenic regions were calculated for tissue samples from human (A), chimpanzee (B) and rhesus macaque (C). The distribution of RPKM values was shown and the percentages of regions with RPKM>0.2 was calculated to estimate the p-values for genomic background transcription with an RPKM cutoff of 0.2. (TIF) [file pgen.1005391.s001.tif]

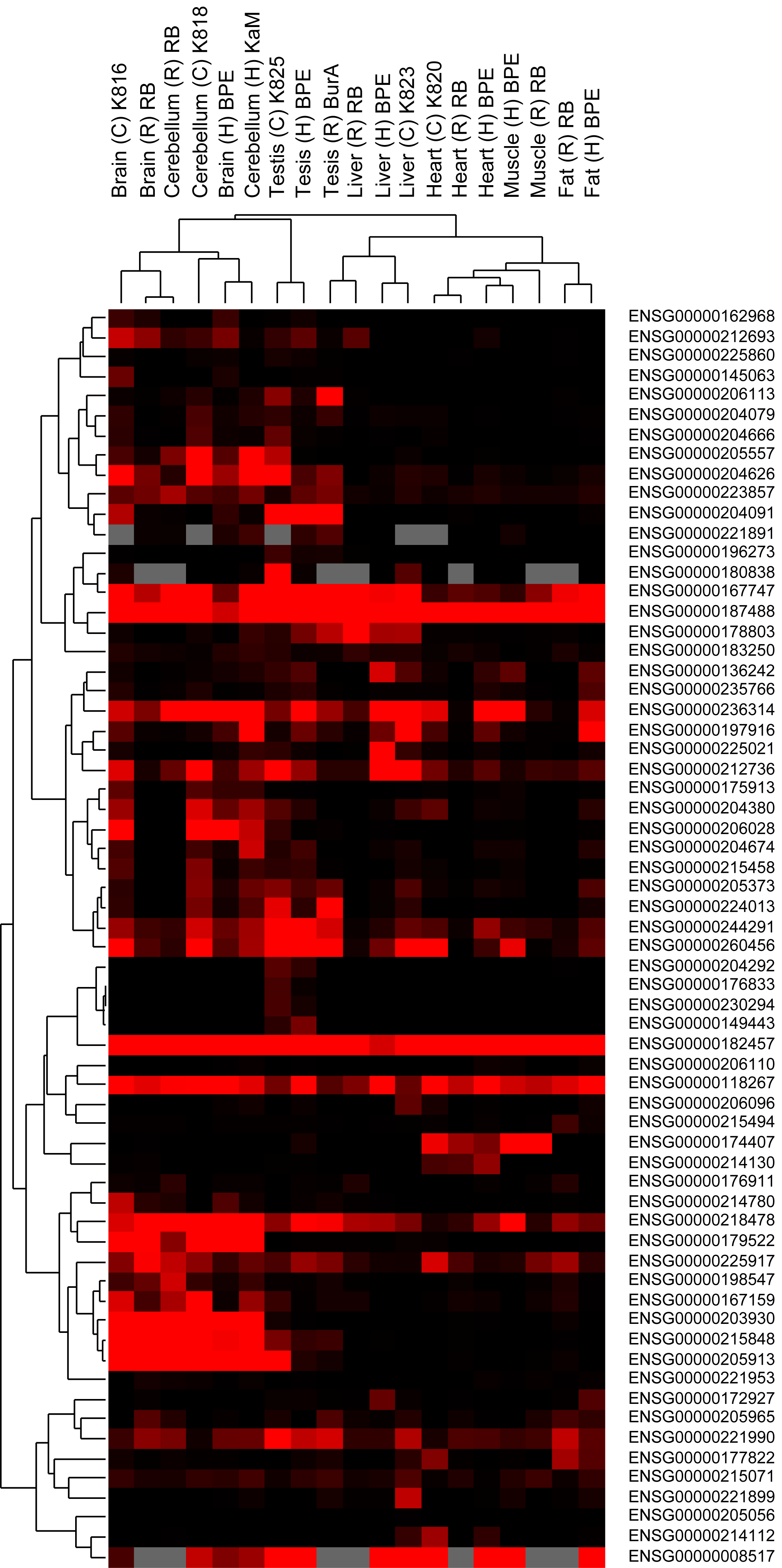

Supplement: S2 Fig — For human de novo genes and their orthologs in chimpanzee and rhesus macaque, the expression levels in different tissues were calculated in terms of RPKM. The RPKM values were then clustered according to similarity using complete linkage hierarchical clustering. Grey boxes: missing data. (TIF) [file pgen.1005391.s002.tif]

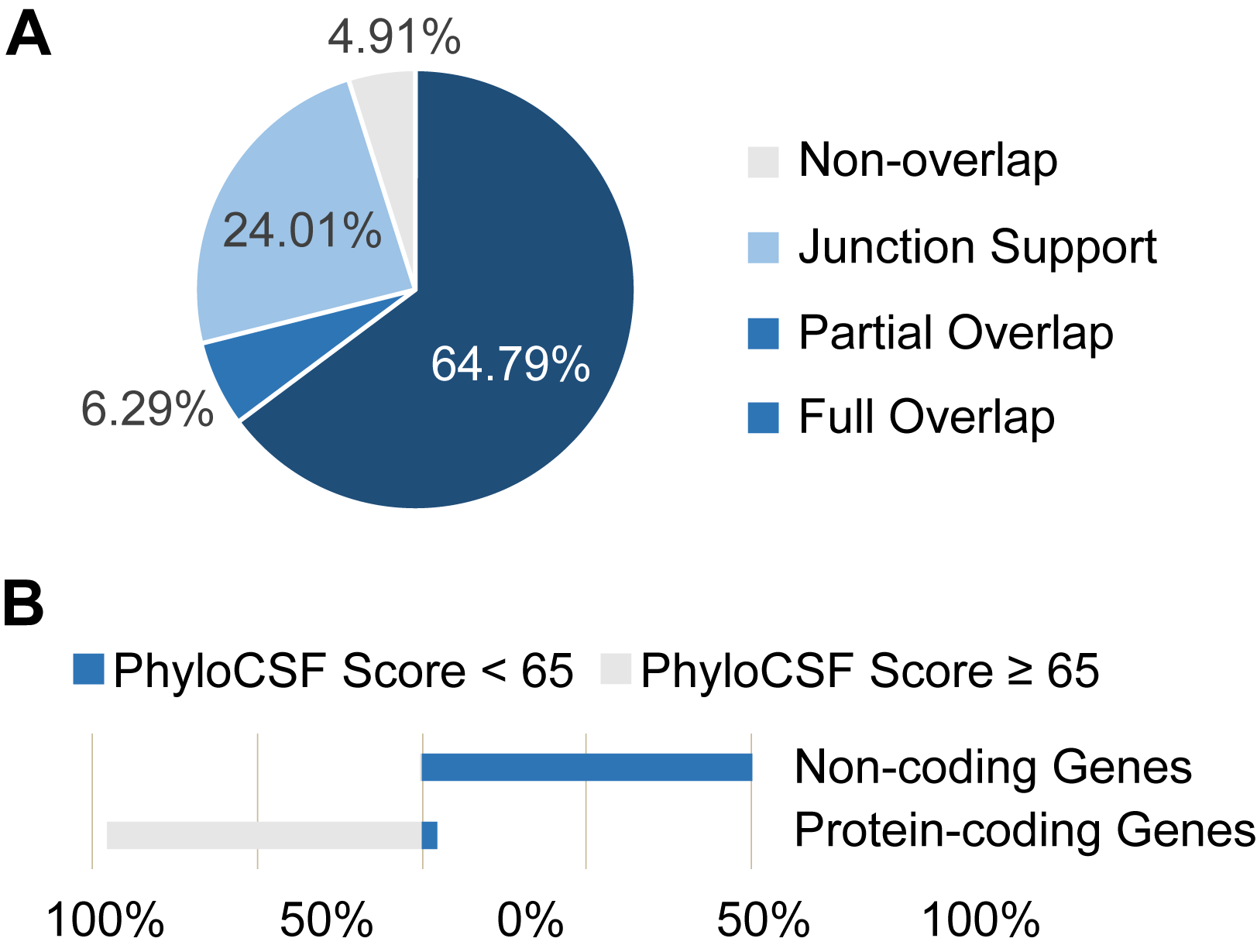

Supplement: S3 Fig — (A) Pie chart showing the percentage of multi-exonic protein-coding transcripts annotated by RefSeq, and reconstructed by our pipeline. The percentages of full overlap, partial overlap and junction support transcripts were calculated and shown. (B) Using PhyloCSF score of 65 as the threshold, the percentage of protein-coding genes and non-coding genes annotated by RefSeq below or above the threshold were shown, respectively. (TIF) [file pgen.1005391.s003.tif]

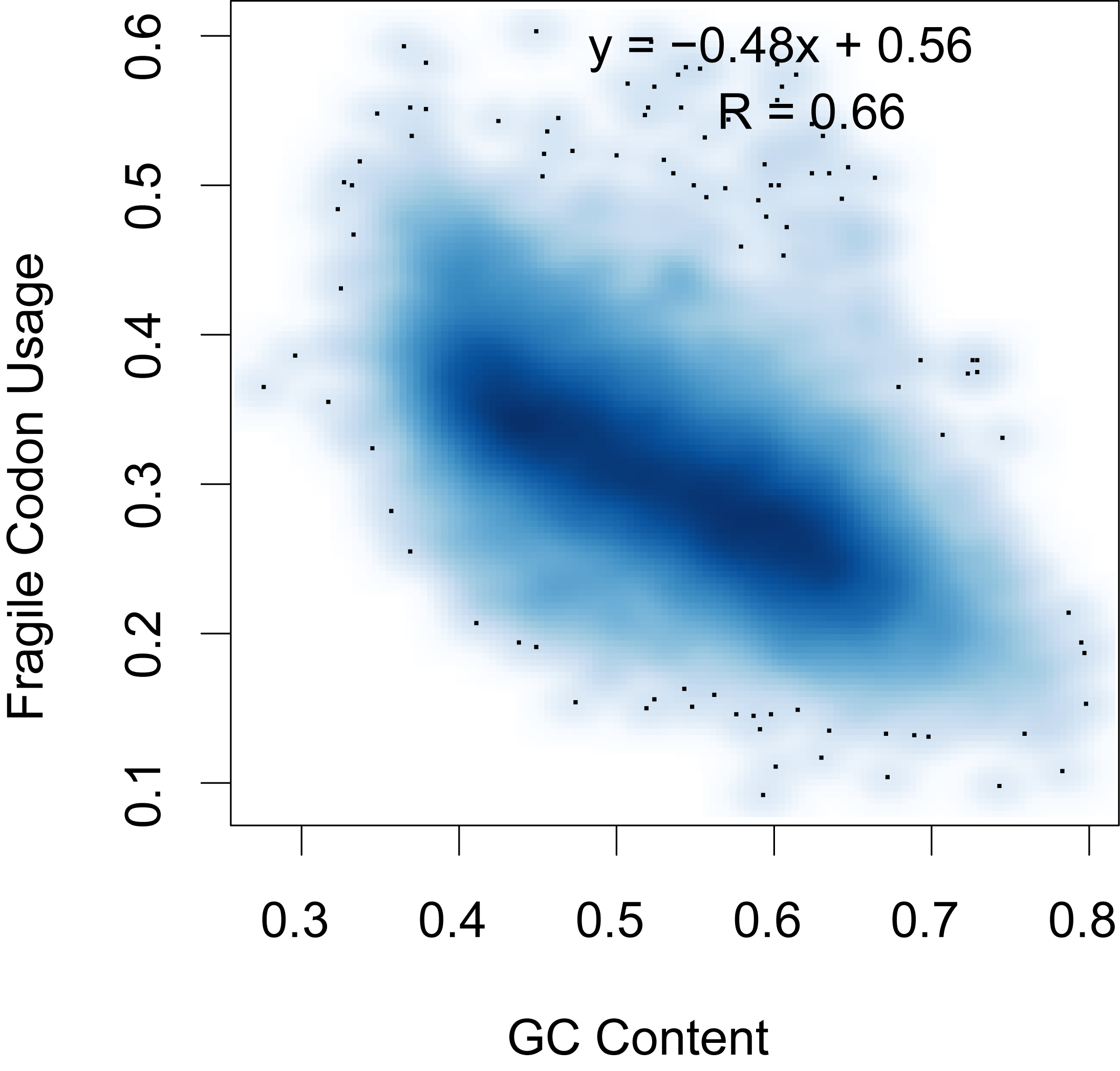

Supplement: S4 Fig — The percentages of fragile codons are plotted against the GC content for known protein-coding genes as annotated by RefSeq. (TIF) [file pgen.1005391.s004.tif]

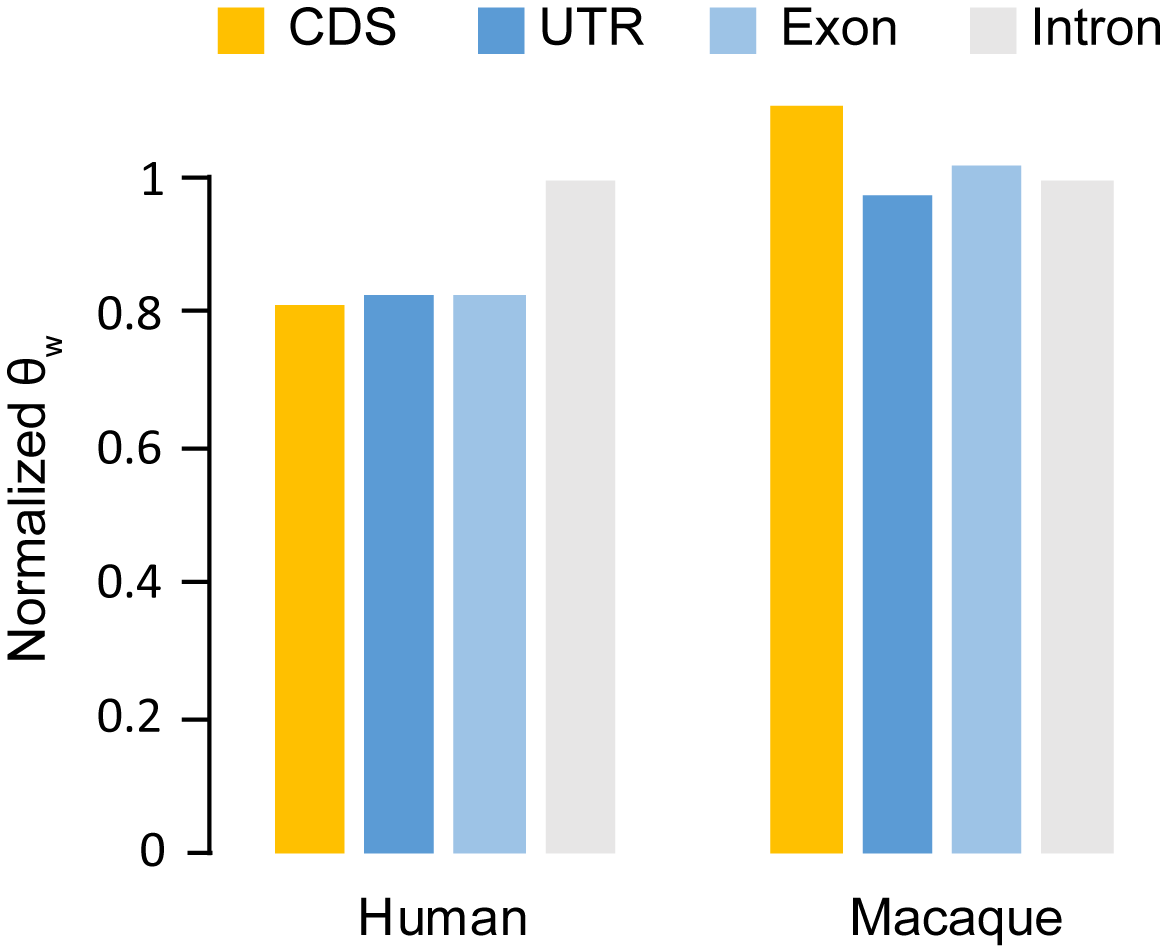

Supplement: S5 Fig — The θ w values were calculated for different regions and then normalized with that of intronic regions. (TIF) [file pgen.1005391.s005.tif]

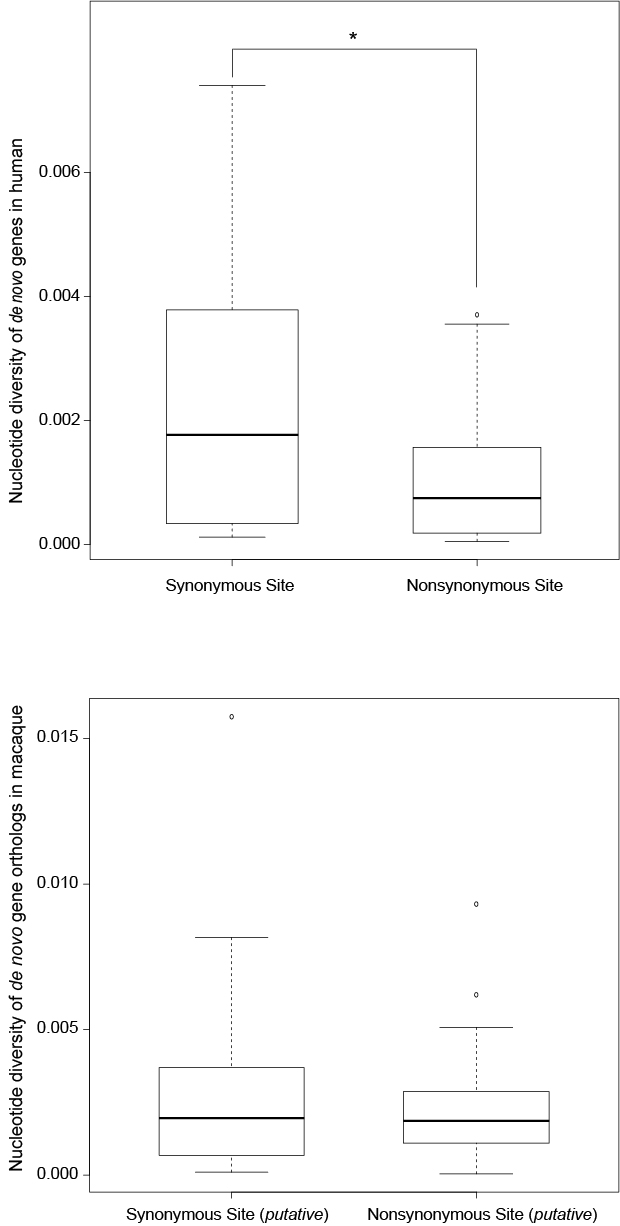

Supplement: S6 Fig — The distributions of nucleotide diversity were summarized as boxplots for human (A) and rhesus macaque (B), respectively. The pseudo-non-synonymous and pseudo-synonymous sites in macaque orthologs were determined by codon-level alignment with human de novo proteins. (JPG) [file pgen.1005391.s006.jpg]

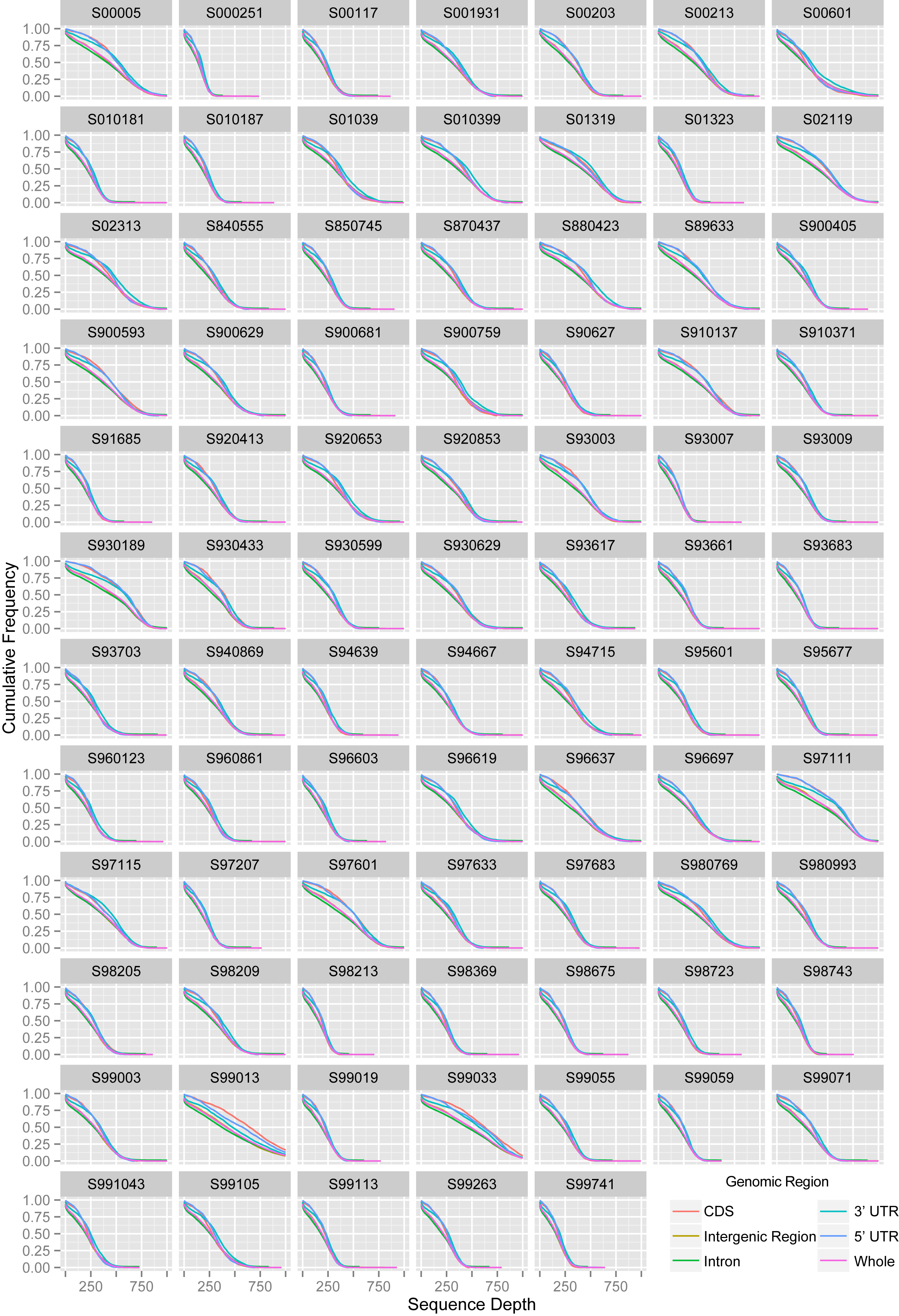

Supplement: S7 Fig — For each macaque sample, cumulative frequency distributions of sequencing coverage for different genomic regions are plotted separately. (TIF) [file pgen.1005391.s007.tif]

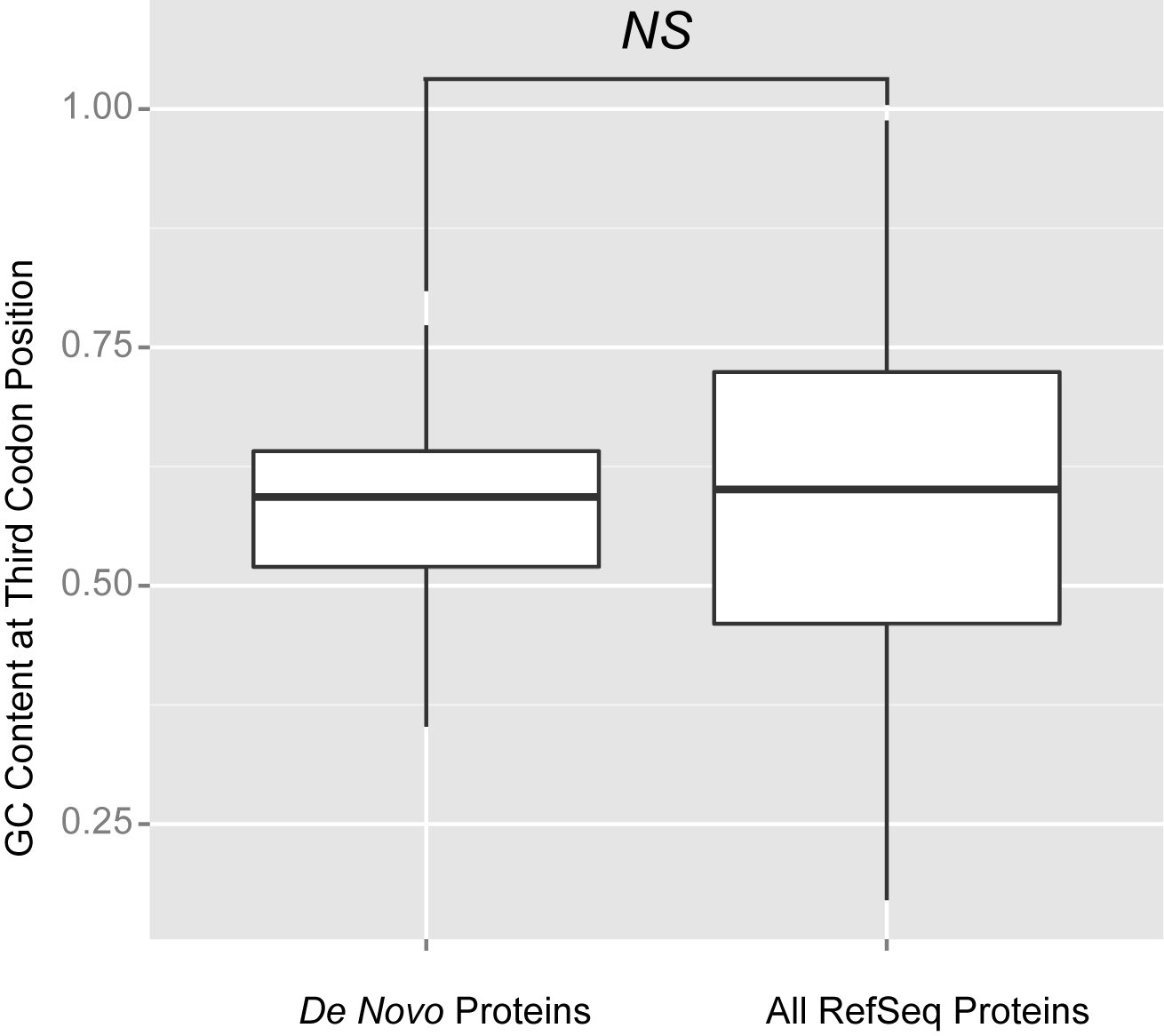

Supplement: S8 Fig — The GC contents at the third codons for de novo genes and known protein-coding genes as annotated by RefSeq are summarized in boxplots, respectively. (TIF) [file pgen.1005391.s008.tif]
