# Supplementary material for: Emergence, Retention and Selection: A Trilogy of Origination for Functional De Novo Proteins from Ancestral LncRNAs in Primates
Source: PLoS Genet. 2015 Jul 15;11(7):e1005391. doi: 10.1371/journal.pgen.1005391 (PMC4503675; doi:10.1371/journal.pgen.1005391)
Supplement: S2 Table — (PDF) [file pgen.1005391.s010.pdf]

S2 Table. Evidence supporting the transcription and translation of *de novo* protein-coding genes in human

Gene ID	EST / mRNA	Iso-Seq	Peptides (ID)*
ENSG00000136242	1		MFMAAAAVAVGAARRPR (PXD000158)<br>SPVSRKMFMAAAAVAVGAAR (PXD000158)<br><u>GQGRGRTAGARARGAPWES (8476)</u><br><u>KMFMAAAAVAVGAAR (8653)</u>
ENSG00000162968	1		APFKELATPLTCKQPPTLK (PXD000158)<br>ELATPLTCKQPPTLKLIR (PXD000158)<br><u>VFHPKGLCCGR (8667)</u><br><u>GLCCGRCSDPR (8667)</u>
ENSG00000175913	2		AHTRTTQYTEQDSVVTAR (PXD000451)<br>EGEGRCEQVALSSGPPEGALHASR (PXD000036)<br><u>PESIFVPTAQDGAQMVCK (PXD000158, 8670)</u><br>PSTGETFVQSGRWDGGWR (PXD000158)<br>PSTGETFVQSGRWDGGWRGAMK (PXD000158)<br>QASTPPTRPESIFVPTAQDGAQMVCK (PXD000343)<br>TTQYTEQDSVVTARGLLDAKR (PXD000158)<br><u>TACNITAWGGEFGK (8670)</u><br><u>HRQASTPPTR (10281)</u>
ENSG00000176833	1		ARSAAKLSQVCVTMCAFICV (PXD000158)<br>ASHSNPSELGGPHSAGFYELLR (PXD000158)<br>ASHSNPSELGGPHSAGFYELLRR (PXD000158)<br>MQMTQRSHHFLVWGGGGRK (PXD000158)<br><u>QSPINVPQSDAGETEKGR (PXD000158, 673)</u><br><u>SAAKLSQVCVTMCAFICV (1624,8661,8664,8670)</u><br><u>LSQVCVTMCAFICV (8669)</u>
ENSG00000176911	1		KDVGWSLGIIPWSSGYPR (PXD000158)<br>LCTCAQAPAAGVPSTAQLFMK (PXD000158)<br>LCTCAQAPAAGVPSTAQLFMKNR (PXD000158)<br>VKPRSPTNTHAHLELTLVK (PXD000158)<br><u>RNAEWHVHMMEYYAAENMNNWSHGMNEAER (203)</u>
ENSG00000180838	1		PPTHGCARSSTAPPAGCSSPPAGSPR (PXD000158)<br>SSTAPPAGCSSPPAGSPR (PXD000158)<br>SSTAPPAGCSSPPAGSPRCSPGLCR (PXD000158)<br><u>CSPGLCRSSTAGQCLWGGVGGASEAAP (156)</u>
ENSG00000187488	3	25	<u>AQEVPALGTASSVAPAR (489)</u>

		KAPRSWDSATPSEMLCPF (8543)
ENSG00000196273	5	IILEKMQSDDVLDGNRER (PXD000158)<br>INMETSKIILEKMQSDDVLDGNR (PXD000036)<br>MGIGTGHTSMNKGGK (PXD000158)<br>MGIGTGHTSMNKGGKDVTLLELSVEK (PXD000158, PXD000451)<br>MQSDDVLDGNRERSNER (PXD000158)<br>YIKTGKSIQVEGTVR (PXD000158)<br>IILEKMQSDDVLDGNR (96, 97)<br>IILEK (3651)<br>MGIGTGHTSMNK (8660)<br>SNEREGRDSLSEK (9330)
ENSG00000197916	1	CAPSHPANVYFFVERVSLTMLPRLVLK (PXD000158)<br>PSSHLSLQSSWEHKCAPSHPANVYFFVER (PXD000158)<br>QGLACGR (8666)
ENSG00000204079	1	GSHGSLGEAPWGKSVPGRGTASR (PXD000158)<br>HQCSGDLEVRGSHGSLGEAPWGK (PXD000158)<br>SLISAWATYQDLISINK (PXD000158)<br>SLISAWATYQDLISINKLKEK (PXD000158)<br>HCMASGAVLNK (8668)<br>MISVHCNLCLPGSSDPPASASQVAGITGVR (8669)
ENSG00000204292	2	MMLSIRVPGQPAALR (8668)<br>RMMLSIR (8668)
ENSG00000204380	3	AIGWHTSAFSPDCSLELR (PXD000158)<br>ILFSNAFLTLAHRQLIR (PXD000158)<br>SAPGPVLSSSLHSPR (PXD000158)<br>RMVPPRPDSLTGLRSSYAS (8538)<br>DLGVSLFPPFPRS (8658, 8670)<br>SAPGPVLSSSLHSPRCLLLLVFNTTYGHR (8670)
ENSG00000205373	1	SLTSEDAGAGLAGALAASSRQPRKLGRCRQD (8543)<br>VPLFRISLTSEDAGAGLAGALAASSR (86628670)<br>MTATLGSRGAAPGGSANSHGPSDPR (86628670)<br>LPSEAGAGGGASPSSR (8672)
ENSG00000205557	1	VGVVLGHLWPVTKVTR (PXD000158)<br>SLVTLSAHQGR (8663)
ENSG00000205965	1	MIGNMKTARPGWSSGK (PXD000158)<br>MWWHLCTHSAVHPPTPSSFK (PXD000158)<br>RSPAPPRASPPGGSR (PXD000158)<br>PLSLWVGLGNWRGGR (8660, 8665)
ENSG00000206028	2	EGDQPPGGAQSSAGLGLVR (PXD000158)<br>PGPVDSDLTANMGQQSGTSVYR (PXD000158)<br>PGPVDSDLTANMGQQSGTSVYRASSPR (PXD000158)

			SEHSTSGQGTSADTHRSR (PXD000158)
			AIPCLRPGPVDSDLTANMGQQSGTSVYR (8666)
			DVILRVQPPTSTGPPLPPLSLVNR (8670)
			PTGLHTHVPEISLVPPTPSGNQTSAHRR (8671)
			VQPPTSTGPPLPPLSLVNR (8671)
ENSG00000206096	1		MRTVFPLPQVGHRPP (PXD000158)
			QNPSEEMVASVQDTNQHGNLHYR (PXD000158)
			VVCSSQLSFFLVTSPSIPLGR (PXD000158)
			TVFPLPQVGHR (8667)
			QNPSEEMVASVQDTNQHGNLHYRMR (8667, 8668)
ENSG00000206110	1		AGGQGTETLEASREK (PXD000158)
			GQKSLIQSQGQALRLK (PXD000158, 1904)
			KSQMLEDASSMLQSSEGK (PXD000158, 8659, 8668, 8669)
			LGRGQKSLIQSQGQALR (PXD000158)
			MFFSIQLHGETFPGFSPR (PXD000158)
			SQMLEDASSMLQSSEGKWL (PXD000158)
			WRAGGQGTETLEASR (PXD000158)
			WRAGGQGTETLEASREK (PXD000158)
ENSG00000206113	1		CSWHPECVSGQALVKEALAGTR (PXD000158)
			DMTSTLRFHPQSTQMR (PXD000158)
			DMTSTLRFHPQSTQMRR (PXD000158)
			EALAGTRDMTSTLRFHPQSTQMR (PXD000158)
			MGRCSWHPECVSGQALVKEALAGTR (PXD000158)
			RVSPGAPPCPTPTLGGILSR (PXD000158)
			RVSPGAPPCPTPTLGGILSREMGPPSPR (PXD000158)
			VSPGAPPCPTPTLGGILSR (PXD000158)
			VSPGAPPCPTPTLGGILSREMGPPSPR (PXD000158)
			VSPGAPPCPTPTLGGILSREMGPPSPRR (PXD000158)
			MGRCSWHPECVSGQALV (3268)
			EMGPPSPR (8653)
			GLPVTWSQLPPR (8653)
ENSG00000212693	1		YPSSGLVSMTGFGDVPDHMKWFMFR (8234)
			CILHLCLPLPTECFAFCASR (8669)
			VCQLYMHSSLNILGFMTDIEIITQMCLTAK (8670)
ENSG00000214780	2		EGKGLVQSHTGTLWAGPLQEGTQGQT (PXD000158)
			GLVQSHTGTLWAGPLQEGTQGQT (PXD000158)
			LRLREGKGLVQSHTGTLWAGPL (8538)
ENSG00000218478	3	4	MYFCGLGVRWGWGR (8667,8664,8667)
			VLSTDPAAHPCAVR (8667)
ENSG00000223857	2	5	AEVGGGPASEEYARRLAGCCPR (PXD000158)
			ARDSGLASGTRAEVGGGPASEEYAR (PXD000158)

			DSGLASGTRAEVGGGPASEEYAR (PXD000158)<br>GSLPSPPAFPPGIATR (PXD000158)<br>PWPLDRGSLPSPPAFPPGIATRATR (PXD000158)<br>SACRARDSGLASGTR (PXD000158)<br>MSGGHSSPEPPR (8653)
ENSG00000224013	4	2	ESLVSLRTQGTHLGLER (PXD000158)<br>GWAGRVSLGMGTASPGSR (PXD000158, 8662)<br>VEGGDAVLRWSSAFSLPQSPGR (PXD000158)<br>YGSVCGFCNKGWAGR (PXD000158)<br>LTGLMAGDR (2026)<br>TQGTHLGLERR (8653)<br>GGEPPGPPRESLVSLR (8660)<br>VSLGMGTASPGSRGGEPPGPPR (8670)
ENSG00000225021	1		AGKSGWLGCSSSLLANSLLTSFFAHFVTK (PXD000158)<br>HTLASSLLISVGHSTK (PXD000158, 8669)<br>MNSLTVMRVWDHSSSVYLEPTSTGTVRQPAISWIYSK (PXD000158)<br>VWDHSSSVYLEPTSTGTVR (PXD000158)
ENSG00000225860	1		AEKETRSQCECALSNQLK (PXD000158)<br>EDSLSHLSGAGCLFSLDIR (PXD000158)<br>EDSLSHLSGAGCLFSLDIRLQGLWLLDSR (PXD000158)<br>MPQWLVASIIPGCVCESVSR (PXD000158)<br>SQCECALSNQLKARLGTNR (PXD000158)<br>TCTNGLPGPSGLQPPTK (PXD000158)<br>VCAVGLPDSEASGLGLR (PXD000158)<br>LQGLWLLDSRTCTNGLPGPSGLQPPTK (8665)
ENSG00000225917	4		MQPGCAVPQSGRLRGSSR (PXD000036，PXD000158)<br>QPLATTVSLLCRFTWGWTSPFQLWR (PXD000036)<br>ELGQLHNRWGRAGEGPR (PXD000036)<br>FTWGWTSPFQLWRVTPQRAWCR (PXD000036)<br>GSSRGPGRSGPGMAAAGGGSAVEPR (PXD000036，PXD000158)<br>SGPGMAAAGGGSAVEPRRGGRHHR (PXD000079)<br>SVLWQFPKHLGKKELGQLHNR (PXD000079)<br>AGAARQVLALLARPRRR (PXD000079)<br>GSSRGPGRSGPGMAAAGGGSA (PXD000079)<br>GPGRSGPGMAAAGGGSAVEPRR (PXD000158，PXD000451)<br>GWRAGGLSTLSFGTGVR (PXD000158)<br>SGPGMAAAGGGSAVEPRR (PXD000158, 8670)<br>CPQSARCPAVRSGPLLQTR (PXD000158)<br>SGPLLQTRCPECAGAAR (PXD000451)<br>MQPGCAVPQSGR (8670)
ENSG00000230294	2		EWSSCLHQASLLWRK (PXD000158)

			GPQAVAGWGDAPALSWSLPR (PXD000158)<br>HLLFSLTCSLMTARLLDMVPR (PXD000158)<br>KHLLFSLTCSLMTAR (PXD000158)<br>TSLSQGPYSQYPSPHHL (PXD000158)<br>WLLLPHPEGHSPAQAPR (PXD000158)<br>AAQCMCWLHRDVPYER (8653)<br>MTSNLGSPGPQR (8653)
ENSG00000235766	2	21	GNPSLPAPPQPVMRR (PXD000158)<br>GNPSLPAPPQPVMRRLTFGPGPAPASDR (PXD000158)<br>RLTFGPGPAPASDRP (PXD000158)<br>TLPASPSAGTPLWGAGHVLGDAGESPLPSHPCPNR (21)
ENSG00000236314	3		AGTGWGQAGLAGSCADPPR (PXD000158)<br>MGAQSGPLPQTEPWPLK (PXD000158)<br>PGAPPSTHNKERDSFR (PXD000158)<br>STQQGFKVSNWFGTQPGPPKETK (PXD000158)<br>VDSEAAAPPAASVSSQAR (PXD000158)<br>VPWEAVGLAVAAVSCSQASPASPGSGRR (PXD000158)<br>VSNWFGTQPGPPKETKAGTGWGQAGLAGSCADPPR (PXD000158)<br>RPGAPPSTHNK (23)<br>AGTGWGQAGLAGSCADPPRVPWEAVGLAVAAVSCSQASPASPGSGR (338)<br>VDSEAAAPPAASVSSQARSGK (8657,8662)
ENSG00000205056	3		FSKLLLKILFYISIFS (PXD000079)<br>ILFYISIFSYPELMCEQYVTFIKPGIHYGQVSKK (PXD000451)
ENSG00000178803	3		EQSQKPCSNGGPAAAGEGR (PXD000158，8663)<br>HTDPNFPSNSNAVGTSSGWNR (PXD000158)<br>IGTGCSHTWDWR (8661,8664)<br>MEQDWQPGEEVTPGPEPCSK (8663)<br>PCSNGGPAAAGEGR (69,73,74,75,76,8653,8661,8664,8667)<br>PCSNGGPAAAGEGRVLPSPCFPWSTCQAAIHK (8663)<br>WQGCTRPALLAPSLATLK (8668,8672)
ENSG00000204626	1		NPHSWGIKAHGLRPPWAPR (PXD000158)<br>RTHSDTQATGWPPPQR (PXD000158)<br>SCPPSLCGGAAQTGDPVALPHGPEK (PXD000079)<br>THSDTQATGWPPPQR (PXD000158)<br>WVWGGGLSPRNPHSWGIK (PXD000158)<br>WQGCTRPALLAPSLATLK (8668; 8672)<br>NPHSWGIKAHGLR (8670a)<br>LERCMVPESEWAPWQPQLPCEPK (8670b)
ENSG00000198547	6		AMGGMPRMGTVGDFGQALSSLAWTSTCFQDFCLPSLPGK (PXD000036, PXD000158)<br>ATSVLAGLTAHLWDLGGGAGRRTSK (PXD000036, PXD000158)

			GDFGQALSSLAWTSTCFQDFCLPSLPGK (PXD000079)<br>GRAMGGMPRMGTVGDFGQALSSLAWTSTCFQDFCLPSLPGK (PXD000036)<br>IWVGGEGWGEVGGLRLSKVGR (PXD000036)<br>LAGLTAHLWDLGGGAGR (PXD000079)<br>LASFPFTKTGMLSRATSVLAGLTAHLWDLGGGAGR (PXD000451)<br>LPAPLISKQQFLSNSSR (PXD000451)<br>MFPRPVLNSRAQAILLPQPPNMLDHR (PXD000451)<br>PNMLDHRQWPPRLASFPFTK (PXD000079)<br>PVLNSRAQAILLPQPPNMLDHR (PXD000036)<br>QPPPPQHPGPYQERIWVGGEGWGEVGGLR (PXD000036)<br>CTSKAQRVHPQPSHQRQ (N.A.)
ENSG00000145063	5		CGKCVKMQILGSTLK (PXD000036)<br>KQVSWIIFLKIGAGCQVHVGHDCSTLR (PXD000158)<br>MHYGRKQVSWIIFLKIGAGCQVHVGHDCS (PXD000079)<br>MQILGSTLKLFRHPPSQ (PXD000079)<br>MQILGSTLKLFRHPPSQVTR (PXD000158)<br>QVSWIIFLKIGAGCQVHVGHDCSTLR (PXD000158)<br>WTLSGSQQTGCVALT (PXD000451)<br>WTLSGSQQTGCVALTVPSFPWVASRMHYGR (PXD000158)<br>WTLSGSQQTGCVALTVPSFPWVASRMHYGRK (PXD000451)<br>LWGRHHLK (8653)<br>MHYGRKQVSWIIFLK (9327)<br>MQILGSTLKLFR (8653)
ENSG00000172927	>10	3	DSLLMFTRQAGHFVEGSKAGR (PXD000036)<br>GVSFLTFHLHQSVPLGDRD (PXD000079)<br>ICVTYTPALPIGLCTR (PXD000158)<br>CCLCLEQSPSWCHCLR (PXD000158, 8653, 8663, 8670)<br>GVSFLTFHLHQSVPLGDRDSLLMFTRQAGHFVEGSK (PXD000451)<br>HGLSMEIMWARMDVALR (human_proteome_map,peptidealtas)<br>ACLTLHRHPTPHCSTWGLPLR (8659)<br>DSLLMFTRQAGHFVEGSK (8666)<br>GAQTGAGLSQEAEDVDVSR (8670)<br>GVSFLTFHLHQSVPLGDR (8653; 8658)<br>MALRICVTYTPALPIGLCTR (8671)<br>MDVALRSPGR (8658)<br>VGVEQAISSCPEEVHGRHGLSMEIMWAR (8658)<br>VTDAPQGTLCGTGNR (8662)
ENSG00000177822	>10	1	ACLGLPSECGSLVRARASPR (PXD000158)<br>GGDWCALSLVAPAASRHPPLSASEQR (PXD000158)<br>HPPLSASEQRIPPTSTCTCTR (PXD000158)<br>RGGDWCALSLVAPAASR (PXD000158)

			ACLGLPSECGSLVRAR (8668)<br>PGLPRPSPTCPWK (8653)<br>RDPSGSFR (8668)
ENSG00000179522	>10	7975	ALGSLSSLMPSFRRSPAALTPGLLR (PXD000158)<br>RSPAALTPGLLRPLAK (PXD000158, 288, 333)<br>SPAALTPGLLRPLAK (PXD000158)<br>VCSPMDAGRGLSLSALTQLR (PXD000158)<br>WGGMLAWPPAAPARALGSLSSLMPSFR (PXD000158)<br>WGGMLAWPPAAPARALGSLSSLMPSFRR (PXD000158)<br>EQAPRVSAPPGR (8665)<br>FLTNKWGGMLAWPPAAPAR (8665)<br>PQSSHLLPPVYPPAPLPPPKDVPLQ (8665)
ENSG00000215071	2		MQICFLALWGGTMAVATSAETALRTSYASLK (8663)<br>SPWGLDNILAPRDVSNYK (10531, 8657)
ENSG00000182457	>10	7	RPEFLSRPSVQTEDAPSK (PXD000158)<br>KETTTEQALPPALLLGIR (8658)<br>PEFLSRPSVQTEDAPSK (8653, 8663, 8669)
ENSG00000174407	1		MDTGSREAAFTRDLPR (PXD000158)<br>PADAQVSPEMSITHKEKENAHLK (PXD000158)<br>DLPRKPADAQVSPEMSITHKEK (PXD000451)<br>EMSITHKEKENAHLKEILLFVNAEAFSQPQPHSAPVCEG (8531)<br>KPADAQVSPEMSITHK (8671)<br>LLCYGWSHC (9328, 9342)
ENSG00000203930	>10		FVDMEFLDQMILR (8662)<br>MPLEKFVDMEFLDQMILR (8670)
ENSG00000204091	>10		HFLGTGKPPHPLGRSIP (PXD000079)<br>SIPVEPCPGLPAFAEVDLLSLLVPIKISSTPPSGSR (PXD000158)<br>ISSTPPSGSR (8653)
ENSG00000204666	3		AIHAAASISCGSIAK (PXD000158)<br>AIHAAASISCGSIAKYVL (PXD000158)<br>ILPRLSDSLMNYPSQSTAGAR (PXD000158)<br>LSDSLMNYPSQSTAGAR (PXD000158)<br>MSLDSGALKDLANGGLDLGSASHAK (PXD000158)<br>APEGDGPLMADAGETLASGGPRVEHTK (8672)<br>SWLILASVCGVEKAIHAAASISCGSIAKYVL (8671)<br>VEHTKSWLILASVCGVEK (8659; 8667)
ENSG00000204674	1		MPLPGTPGPVTTSPQTPTPR (PXD000036, PXD000158)<br>ILSGKGSGGSARAVSK (PXD000158)<br>MPLPGTPGPVTTSPQTPTPRPLTTDWRILSGK (PXD000158)<br>SQEEAAPPSPRPQSR (PXD000158)<br>AHAQTTNPYWADTNTR (9299)

			<u>ILSGKGSGGSAR (8653)</u>
			<u>KSQEEAAPPSPRPQSR (9331)</u>
			<u>LRSSSSGNSLLR (8653, 9333)</u>
			<u>PLTTDWRILSGK (8661, 8664)</u>
			<u>RILSGKGSGGSARAVSK (8409)</u>
ENSG00000212736	1		AWQLSGSPSSYRQGSGVK (PXD000158)
			AWQLSGSPSSYRQGSGVKWTVK (PXD000158)
			CCDLPAISPFLSQPR (PXD000158)
			<u>MLNWLAQIQQIAKSGR (PXD000158, 8659)</u>
			MLNWLAQIQQIAKSGRAWQLSGSPSSYR (PXD000158)
			SGRAWQLSGSPSSYR (PXD000158)
			SGRAWQLSGSPSSYRQGSGVK (PXD000158)
			VQLLLDGGTFQSLVR (PXD000158)
ENSG00000167747	>10	40	ETWAWLSRTDTAWPGAPGVKQAR (PXD000158)
			LGEEPPPLPYCDQAYGEELSIR (PXD000158)
			LLSMVPGPARPPGSCWDPTQCTR (PXD000036)
			<u>MTVLEAVLEIQAITGSR (PXD000158, 8666)</u>
			MTVLEAVLEIQAITGSRLLSMVP (PXD000079)
			MTVLEAVLEIQAITGSRLLSMVPGPAR (PXD000036)
			PPGSCWDPTQCTRTWLLSHTPR (PXD000036)
			WISGLPRASCRLGEEP (PXD000079)
			TVLEAVLEIQAITGSR (human_proteome_map,peptidealtas)
			<u>ASCRLGEEPPPLPYCDQAYGEELSIR (9335)</u>
			<u>TDTAWPGAPGVKQAR (608)</u>
ENSG00000214112	>10		ATETKENFQKESMVK (PXD000036, PXD000451)
			SEQLGTGGDQDWQLQGHTR (PXD000158, PXD000451)
			<u>LGRQIQPPWPLAHAR (8665)</u>
ENSG00000214130	6		<u>AAAFLHAAQGSSMPGAGMR (3669, 3674, 8653)</u>
			<u>EVSLDSR (2674)</u>
			<u>FCSLLAILASPNERALK (8660, 8663, 8669)</u>
			<u>LAPPRR (8653)</u>
			<u>MAMAHAGLCGWR (8653)</u>
ENSG00000118267	>10	11	AFSQCSALTLHQRIHTGKK (PXD000036, PXD000158)
			AFSTCTDLIEHQKTHAEEK (PXD000158)
			APTLFFIRESTLERNLMHVVIVLK (PXD000036)
			AQTSLNIREYTLVKSLIIAIVVR (PXD000158)
			CDACGKAFSTCTDLIEHQKTHAEEK (PXD000158)
			CLVEDEILLNITEFIQVRNPMNVMNVGK (PXD000036)
			CLVEDEILLNITEFIQVRNPMNVMNVGKPLVR (PXD000036)
			IQTLLNIEGSTLERNPINVMSVGK (PXD000158)
			NHMHVISVIKVLVKAQTSLNIR (PXD000158)

		NLMHVVIVLKALVAVQILLSIK (PXD000158，8658)
		NLMHVVIVLKALVAVQILLSIKEYTLER (PXD000451)
		NPMNVMNVGKPLVRAPTLFFIR (PXD000036)
		NYYLCTQCSKSFSQISDLIK (PXD000036)
		PSVGFQILLIINEFTLER (PXD000158, 8658)
		SDLINHQKIHTGEKPYK (PXD000158)
		SLTHVISAIKCLVEDEILLNITEFIQVRNPMNVMNVGK (PXD000036)
		THAEEKPYQCVQCSR (PXD000158，8658)
		VRKPLVCTPTLFSTRDTVPEKNLMNAVDY (PXD000079，8665)
		VSVRIQTLLNIEGSTLERNPINVMSVGK (PXD000036，1805)
		YYLCTQCSKSFSQISDLIK (PXD000036, PXD000158，1831)
		AFSTCTDLIEHQK (8658，1789; 1828; 1830; 1831)
		CDACGKAFSTCTDLIEHQK (2027,8665)
		ECGKSFSR (1805,8660)
		HQRIHTGK (1831,8663)
		IHTGEKPY (1789, 1828, 1830, 1831，8663; 8665; 8671)
		IHTGKK (2027,8658)
		NPIPVINVAK (8660)
		PLVCTPTLFSTRDTVPEKNLMNAVDY (8663)
		PNPCDECGKSFSR (8663, 8665, 8671)
		PSVRAQILFCIR (8658)
		PYKCDACGKAFSTCTDLIEHQK (9308)
		QKIHTGEK (76)
		SDLIKHQR (1824)
		AAGPGGTGQAECQVVVATHR (PXD000158)
		AAGPGGTGQAECQVVVATHRANLKPWDGR (PXD000036)
		DANTNRKGPMHGSDFPPR (PXD000158)
		DHQGSVEDTSLGGDAPADGVSPSVPPLQGLG (PXD000079)
		DHQGSVEDTSLGGDAPADGVSPSVPPLQGLGK (PXD000158, 146)
		ETRSGGPRGPRHSAP (PXD000079)
		KGPMHGSDFPPRLCLHSMSLWGSWGSR (PXD000036)
		LALQGPPGTILSLSSSSPCLPPSHCDPG (PXD000079)
ENSG00000215458	4	LCLHSMSLWGSWGSR (PXD000158)
		LCLHSMSLWGSWGSRPPSPAHSREVAS (PXD000451)
		LLLQVSFQGAADGCSLR (PXD000036, PXD000158)
		PRDHQGSVEDTSLGGDAPADGVSPSVPPLQGLG (PXD000079)
		RTEEGRLSTSSCASVSR (PXD000158)
		LSTSSCASVSR (8663)
		PMHGSDFPPR (8385; 8417)
		PPSPAHSREVAS (9340)
		RASRLALQGPPGTILSLSSSSPC (8538)

			RSGGPR (8665)
ENSG00000215494	1		GDSEQSASKSKQAFPLLLR (PXD000158)
			GGGQTGTFHMGLLPSR (PXD000158)
			GMEWPLSAWLESSSPAPCMPLWRR (PXD000158)
			HLTGVARGGGQTGTFHMGLLPSR (PXD000158)
			LTQRHLTGVARGGGQTGTFHMGLLPSR (PXD000158)
			MPSQPHLSACSVESPGAAAR (PXD000158)
			MPSQPHLSACSVESPGAAARLTQR (PXD000158, 8669)
			QECAPVSALTLTTTMGR (PXD000158)
			QECAPVSALTLTTTMGRLFSVHWQIWGPCSPCIVYPK (PXD000451)
			KGMEWPLSAWLESSSPAPCMPLWR (8661; 8664; 8669; 8670)
			LFSVHWQIWGPCSPCIVYPKGDSEQSASK (8663; 8669; 8670)
			NSFWESRPSPCR (8653)
ENSG00000215848	>10		GDCVLEVMLHLLSSAQHSGETASSQR (PXD000158)
			GDCVLEVMLHLLSSAQHSGETASSQRPLTAPSTK (PXD000158)
			GGREASALGLVPRLLR (PXD000158)
			KVLEESEWGNISMRR (PXD000158)
			VLEESEWGNISMRRER (PXD000158)
			PLTAPSTK (76)
ENSG00000221953	2		AAVGATPTTGPGTGGEGALLGCGSGR (PXD000079)
			AGPDPTPSPAPPPAGPR (PXD000343)
			AGPDPTPSPAPPPAGPRPSPGSLGPSAPAAPR (PXD000158)
			AGPDPTPSPAPPPAGPRPSPGSLGPSAPAAPRTAR (PXD000158)
			GAYELQGGASQDGPGQAAV (PXD000079)
			GCRGHFRSLPAAASR (PXD000158)
			GQRGGSGGCQSTRAAER (PXD000036, PXD000158)
			KSSTSCGTWTASGLPSLGHLPR (PXD000158)
			KSSTSCGTWTASGLPSLGHLPRR (PXD000036, PXD000158)
			LLPAEVPPGAAAANFPERER (PXD000079)
			MGLCTLQPLGPPRKSSTSCGTWTASGLPSLGHLPR (PXD000451)
			RLLPAEVPPGAAAANFPERE (PXD000079)
			SLPAAASRSGSRTLR (PXD000158, PXD000451)
			SSTSCGTWTASGLPSLGHLPRR (PXD000158, 8671)
			MGLCTLQPLGPPRK SSTSCGTWTASGLPSLGHLPRR (8667)
ENSG00000221891	3	4	FSACPVSRSWCPERNFSGSIPAVTPPK (PXD000079)
			HGSVSLLRHPGTTTFVQQR (PXD000158)
			IVFSACPVSRSWCPER (PXD000036)
			IVFSACPVSRSWCPERNFSGSIPAVTPPK (PXD000158)
			LPGHSKSEGPPGKVR (PXD000158)
			NFSGSIPAVTPPKLPGHSKSEGPPGK (PXD000036, PXD000451)
			PGPSAWRASPAGWAGRAKLR (PXD000079)

			PYTLTGGRHGSVSLLRHPGTTTFVQQR (PXD000158)<br>RNFSGSIPAVTPPKLPGHSKSEGPPGK (PXD000079)<br>GSCPLLPGPSAWR (8666)<br>MEGCAVR (8653)<br>MEGCAVRRGSCPLLPGPSAWR (9297, 9304, 9305, 9306, 9334, 9335, 9336, 9337, 9338)<br>SWCRASGLPNR (9353)
ENSG00000221899	2		MNTYTRSASFTPKTFTFPLAQNR (PXD000036, PXD000158)<br>AECTAKLAGDGHARLASAFLHR (PXD000158)<br>DSLPPLGSLTLGKSPK (PXD000158, 1789)<br>LGEGGSAPALGPSPRRGR (PXD000158)<br>PRLGEGGSAPALGPSPR (PXD000158)<br>TSHPTTTSKLKYLGR (PXD000158)<br>VSEGLRDSLPPLGSLTLGKSPK (PXD000158)<br>AALPPPLGTCRPR (1789)<br>DSLPPLGSLTLGK (2001; 9330)<br>LASAFLHR (10049)<br>MNTYTRSASFTPK (1789)<br>NTYTRSASFTPK (1789)<br>RAALPPPLGTCR (8653)<br>RAALPPPLGTCRPR (1789)<br>VSEGLRDSLPPLGSLTLGK (1789)<br>YLGRPSPR (96)<br>YLGRPSPRR (9333)
ENSG00000260456	>10	16	RCHHHHEATGAASGAAAGGPGAGCVGLCRLAL (PXD000079)<br>FGGRLPVSRVEAALPYWVPL (PXD000079)<br>KEVCLPRHSMHPGPWAICC (PXD000079)<br>LALTPSAQDGRNSTFQTYKK (PXD000158)<br>LPVSRVEAALPYWVPLSLR (PXD000158)<br>NSTFQTYKKEVCLPR (PXD000451)
ENSG00000149443	9		YPRETETIWSHPYVVEGSHK (PXD000036)<br>EKGVAQRQVPTTGTHSFFHCTSEGNK (PXD000036)<br>IHTPEQGSPSLGQNWSWGNRKEK (PXD000036)<br>SLHGCLAAMAPSITSSEDSSPSQRDK (PXD000079)<br>EKGVAQRQVPTTGTHSFFHCT (PXD000079)<br>ETETIWSHPYVVEGSHK (PXD000158, PXD000451)<br>GVAQRQVPTTGTHSFFHCTSEGNK (PXD000158)<br>IHTPEQGSPSLGQNWSWGNR (PXD000158)<br>KRSQGLHWSHWQGLNHMSIR (PXD000158,human_proteome_map,peptidealtas)<br>QVPTTGTHSFFHCTSEGNKEK (PXD000158)

			SQGLHWSHWQGLNHMSIR (PXD000158)
ENSG00000167159	1		AASGDSQEGMRRGYEER (PXD000158)
ENSG00000008517	>10	10	GDKEELTPQKCSEPQSSK (PXD000158)<br>QFQSFCCSLSELFMSSFQSYGAPRGDKEELTPQK (PXD000158)<br>SYGAPRGDKEELTPQK (PXD000158)
ENSG00000183250	8	1	SKINPPPWAPACLPGGFPACLPAPK (PXD000036)<br>SKINPPPWAPACLPGGFPACLPAPKSSTDSASSCFK (PXD000036)<br>GRSKINPPPWAPACLPGGFPACLPAPK (PXD000036)<br>MGWDCRRTTVENPSPIRN (PXD000079)<br>MGWDCRRTTVENPSPIRNC (PXD000079)<br>EFSDPLDIPGAGAMG (PXD000158)<br>MGWDCRRTTVENPSPIR (PXD000158, PXD000451)
ENSG00000244291	4	73	MLANHALSTPHCACSPAPAPR (PXD000036)<br>PTLRMLANHALSTPHCACSPAPAPR (PXD000036, PXD000158)<br>SHAPQPPAHLGLGPGCFPAVAVVVPVPGSRAHR (PXD000036)<br>GDPPIPPRRSGVLAR (PXD000079)<br>MLANHALSTPHCACSP (PXD000079)<br>MLANHALSTPHCACSPAPA (PXD000079)<br>MLASPARPTLRMLANHALSTPHCACSPAPAPR (PXD000158, PXD000451)<br>SHAPQPPAHLGLGPGCFPAVAVVVPVPGSR (PXD000158)<br>CVPVEARAAGVFGDRLAGVFGSR (PXD000158)
ENSG00000205913	5		MSMAACPEAPEPSFLR (PXD000036)<br>EVPSSPASTQWHRPCNFR (PXD000036)<br>MSMAACPEAPEPSFLREVPSSPASTQWHR (PXD000158)<br>NLVWRDVSLGQTSRTPR (PXD000158)
ENSG00000221990	2	14	MFPGPLRGPAAQVLENECGSLGRAAEGR (PXD000036)<br>MPAVFMLASSSALQCGRGVPR (PXD000036)<br>MPAVFMLASSSALQCGRGVPRFPR (PXD000036)<br>GNNASGMGGHRMFPGPLR (PXD000036, PXD000158)<br>QERSHWHPRGNNASGMGGHRMFPGPLR (PXD000079)<br>RGNNASGMGGHRMFPGPLR (PXD000079)<br>AEKVGNQTSVIPATSRQAAL (PXD000079)<br>MLASSSALQCGRGVPRFPR (PXD000079)<br>MPAVFMLASSSALQCGRGV (PXD000079)<br>AEKVGNQTSVIPATSR (PXD000158)<br>FPRTEVGAGHSVNEETK (PXD000036, PXD000158)<br>MPAVFMLASSSALQCGR (PXD000158)<br>SHWHPRGNNASGMGGHR (PXD000158)<br>TEVGAGHSVNEETKAEK (PXD000158)<br>TEVGAGHSVNEETKAEKVGNQTSVIPATSR (PXD000036, PXD000158)

*peptides with underline were previously reported.
