# Supplementary material for: Emergence, Retention and Selection: A Trilogy of Origination for Functional De Novo Proteins from Ancestral LncRNAs in Primates
Source: PLoS Genet. 2015 Jul 15;11(7):e1005391. doi: 10.1371/journal.pgen.1005391 (PMC4503675; doi:10.1371/journal.pgen.1005391)
Supplement: S4 Table — (PDF) [file pgen.1005391.s012.pdf]

S4 Table. Statistics of polymorphic sites in rhesus macaque

	Coding Genes (RefSeq)	Coding Genes (Ensembl)	LncRNAs (This Study)	MiRNAs (miRBase)	Whole Genome
**Length**	11,141,075	33,860,826	3,543,258	43,036	2,864,106,071
**SNP Number**	103,157	339,282	59,856	638	44,356,394
