# Supplementary material for: Emergence, Retention and Selection: A Trilogy of Origination for Functional De Novo Proteins from Ancestral LncRNAs in Primates
Source: PLoS Genet. 2015 Jul 15;11(7):e1005391. doi: 10.1371/journal.pgen.1005391 (PMC4503675; doi:10.1371/journal.pgen.1005391)
Supplement: S5 Table — (PDF) [file pgen.1005391.s013.pdf]

S5 Table. Information of 67 human individuals with whole genome re-sequencing data

Sample ID	Population	Sex	Coverage
**HG02095**	ACB	Female	10.78
**HG02107**	ACB	Male	40.28
**HG02332**	ACB	Male	17.82
**HG02419**	ACB	Female	13.21
**HG02484**	ACB	Male	12.5
**HG02508**	ACB	Female	11.04
**HG02537**	ACB	Female	17.45
**HG02283**	ACB	Male	17.65
**HG02479**	ACB	Female	9.78
**HG02485**	ACB	Female	10.64
**NA19904**	ASW	Male	8.91
**NA19909**	ASW	Female	6.64
**NA19921**	ASW	Female	14.94
**NA19923**	ASW	Female	10.39
**NA19984**	ASW	Male	11.16
**NA19914**	ASW	Female	17.84
**NA12044**	CEU	Female	13.96
**NA12812**	CEU	Male	10.38
**NA12873**	CEU	Female	9.73
**NA12874**	CEU	Male	11.27
**NA12872**	CEU	Male	9.64
**NA12414**	CEU	Female	12.45
**NA18525**	CHB	Female	12.35
**NA18647**	CHB	Male	9.99
**NA18747**	CHB	Male	9.93
**NA18749**	CHB	Male	9.81
**HG00097**	GBR	Female	10.26
**NA21106**	GIH	Female	10.54
**NA21119**	GIH	Male	10.6
**NA18957**	JPT	Female	9.13
**NA18960**	JPT	Male	13.44
**NA18968**	JPT	Female	11.07
**NA18971**	JPT	Male	12.55
**NA18974**	JPT	Male	12.76
**NA18975**	JPT	Female	10.69
**NA18976**	JPT	Female	13.52
**NA18998**	JPT	Female	10.83
**NA19001**	JPT	Female	9.89
**HG02141**	KHV	Male	10.47
**NA19038**	LWK	Female	10.62

**NA19307**	LWK	Male	11.68
**NA19310**	LWK	Female	10.18
**NA19031**	LWK	Male	10.56
**NA19308**	LWK	Male	11.76
**NA19740**	MXL	Female	9.9
**HG02089**	PEL	Female	11.02
**HG02104**	PEL	Male	11.09
**HG02301**	PEL	Female	11.9
**HG02286**	PEL	Female	10.22
**HG00734**	PUR	Female	10.29
**HG01049**	PUR	Female	7.62
**HG01075**	PUR	Male	17.01
**HG01167**	PUR	Male	17.28
**HG01176**	PUR	Male	17.28
**HG00732**	PUR	Female	30.95
**NA18504**	YRI	Male	12.19
**NA18856**	YRI	Male	10.34
**NA18868**	YRI	Male	11.16
**NA19095**	YRI	Female	9.33
**NA19119**	YRI	Male	12.56
**NA19131**	YRI	Female	11.54
**NA19152**	YRI	Female	13.64
**NA19171**	YRI	Male	10.81
**NA19184**	YRI	Male	10.02
**NA19204**	YRI	Female	11.33
**NA19146**	YRI	Male	10.67
**NA20534**	TSI	Male	8.46
