# Supplementary material for: Emergence, Retention and Selection: A Trilogy of Origination for Functional De Novo Proteins from Ancestral LncRNAs in Primates
Source: PLoS Genet. 2015 Jul 15;11(7):e1005391. doi: 10.1371/journal.pgen.1005391 (PMC4503675; doi:10.1371/journal.pgen.1005391)
Supplement: S6 Table — (PDF) [file pgen.1005391.s014.pdf]

S6 Table. Statistics of targeted sequencing in 82 macaque animals

ID	Total Reads	Mapped Reads	Percentage	Uniquely Mapped Reads	Percentage
**00005**	3,967,630	3,493,397	0.88	3,172,619	0.80
**00203**	2,902,460	2,552,595	0.88	2,283,422	0.89
**00213**	3,346,736	2,946,690	0.88	2,662,072	0.90
**01319**	3,615,358	3,165,257	0.88	2,877,326	0.91
**89633**	3,334,400	2,898,002	0.87	2,648,849	0.91
**93003**	3,255,580	2,859,293	0.88	2,601,769	0.91
**930189**	3,687,168	3,077,532	0.83	2,844,214	0.92
**930433**	2,807,842	2,455,930	0.87	2,218,608	0.90
**96637**	3,007,048	2,467,821	0.82	2,261,346	0.92
**97111**	3,864,180	3,228,185	0.84	2,987,302	0.93
**97115**	3,230,670	2,806,632	0.87	2,548,264	0.91
**97601**	3,825,904	3,342,340	0.87	2,994,546	0.90
**00117**	1,936,186	1,508,141	0.78	1,386,288	0.92
**001931**	2,996,308	2,329,887	0.78	2,147,724	0.92
**00601**	2,446,396	2,017,023	0.82	1,890,488	0.94
**01039**	2,876,064	2,307,959	0.80	2,142,556	0.93
**90627**	2,120,884	1,713,951	0.81	1,585,474	0.93
**93617**	2,310,886	1,829,410	0.79	1,696,354	0.93
**93703**	2,247,678	1,781,817	0.79	1,638,244	0.92
**94639**	1,985,094	1,427,599	0.72	1,317,409	0.92
**94715**	2,609,252	2,112,639	0.81	1,951,751	0.92
**96603**	1,717,350	1,368,420	0.80	1,260,959	0.92
**97207**	1,690,180	1,345,900	0.80	1,243,318	0.92
**97633**	2,435,508	1,908,940	0.78	1,759,027	0.92
**98205**	2,166,608	1,725,912	0.80	1,596,058	0.92
**98723**	2,079,812	1,514,537	0.73	1,390,059	0.92
**98743**	1,685,634	1,391,001	0.83	1,291,316	0.93
**99105**	2,445,736	2,023,720	0.83	1,870,347	0.92
**10187**	1,941,136	1,483,225	0.76	1,356,659	0.91
**870437**	2,906,412	2,176,409	0.75	1,980,973	0.91
**900629**	3,009,866	2,345,362	0.78	2,162,773	0.92
**900681**	1,838,262	1,409,905	0.77	1,292,936	0.92
**91685**	2,042,422	1,543,183	0.76	1,410,387	0.91
**920853**	3,026,874	2,291,763	0.76	2,103,438	0.92
**93009**	2,480,674	1,856,959	0.75	1,696,468	0.91
**930629**	2,899,280	2,288,576	0.79	2,107,339	0.92
**93661**	2,191,744	1,689,596	0.77	1,552,145	0.92
**940869**	2,602,128	2,100,929	0.81	1,932,527	0.92
**96619**	3,190,632	2,464,213	0.77	2,269,020	0.92
**98209**	3,110,808	2,309,528	0.74	2,118,886	0.92

**99003**	2,824,394	2,140,918	0.76	1,960,503	0.92
**99071**	3,006,116	2,251,283	0.75	2,059,135	0.91
**99113**	2,093,778	1,542,484	0.74	1,401,820	0.91
**00251**	1,692,044	1,252,288	0.74	1,134,843	0.91
**010181**	2,264,524	1,682,030	0.74	1,536,597	0.91
**01323**	2,104,338	1,574,683	0.75	1,431,340	0.91
**930599**	2,538,342	1,862,743	0.73	1,711,749	0.92
**95601**	2,611,720	1,953,733	0.75	1,779,484	0.91
**95677**	2,242,624	1,703,194	0.76	1,558,492	0.92
**960123**	2,347,346	1,746,902	0.74	1,592,714	0.91
**97683**	2,622,344	1,946,206	0.74	1,771,423	0.91
**98213**	1,676,132	1,246,892	0.74	1,133,964	0.91
**98369**	2,051,034	1,548,186	0.75	1,418,688	0.92
**98675**	1,948,208	1,448,337	0.74	1,324,923	0.91
**99019**	2,045,396	1,483,868	0.73	1,357,644	0.91
**99055**	2,439,512	1,835,777	0.75	1,683,198	0.92
**99741**	1,676,558	1,268,032	0.76	1,151,882	0.91
**840555**	2,368,254	1,811,538	0.76	1,673,449	0.92
**850745**	2,281,744	1,725,307	0.76	1,575,147	0.91
**900405**	2,331,038	1,807,203	0.78	1,667,362	0.92
**910371**	2,422,998	1,807,414	0.75	1,659,654	0.92
**920413**	2,576,346	1,943,167	0.75	1,789,083	0.92
**93007**	1,662,204	1,262,134	0.76	1,158,879	0.92
**93683**	1,980,990	1,492,241	0.75	1,367,582	0.92
**94667**	2,459,184	1,882,834	0.77	1,740,114	0.92
**960861**	2,501,580	1,864,396	0.75	1,701,180	0.91
**96697**	2,673,052	2,131,688	0.80	1,975,016	0.93
**980993**	2,494,096	1,881,584	0.75	1,726,037	0.92
**99059**	2,033,558	1,523,642	0.75	1,398,853	0.92
**991043**	2,295,734	1,707,648	0.74	1,560,410	0.91
**99263**	2,260,332	1,690,204	0.75	1,545,354	0.91
**900593**	3,892,048	3,152,088	0.81	2,925,644	0.93
**900759**	2,985,770	2,513,876	0.84	2,316,709	0.92
**910137**	3,316,194	2,802,118	0.84	2,598,734	0.93
**920653**	3,036,388	2,526,636	0.83	2,330,333	0.92
**99013**	4,233,824	3,142,547	0.74	2,942,323	0.94
**99033**	4,263,558	3,389,478	0.79	3,171,206	0.94
**010399**	5,543,420	3,950,012	0.71	3,599,965	0.91
**02119**	4,192,580	3,117,326	0.74	2,859,913	0.92
**02313**	4,348,838	3,306,464	0.76	3,006,325	0.91
**880423**	4,152,862	3,075,796	0.74	2,806,304	0.91
**980769**	4,307,020	3,232,228	0.75	2,940,933	0.91
**Total**	222,632,812	174,787,303	0.79	160,296,168	0.92
